# Preliminary Synthesis and Basic Performance Characterization of Bio-Based Composite Materials Using Starch and Gelatin as Representative Matrix Phases: Exploratory Conceptual Design for Sustainable Toy Scenarios

**DOI:** 10.3390/polym18192411

**Published:** 2026-10-02

**Authors:** Lijuan Mao, Yuxin Gong, Chenyang Liu, Zhenpeng Li

**Affiliations:** 1 College of Art and Communication, China Jiliang University, Hangzhou 310018, China; p24131357021@cjlu.edu.cn (L.M.); p24131357009@cjlu.edu.cn (Y.G.); 2 Department of Industrial Design, School of Mechanical Engineering, Beijing University of Science and Technology, Beijing 100083, China; liuchenyang0211@gmail.com

**Keywords:** starch-based composites, gelatin-based composites, bio-based composites, sustainable exploratory design, conceptual toy design

## Abstract

The overconsumption of traditional plastic products causes serious white pollution and ecological pressure. Bio-based composites derived from natural polymers have emerged as promising sustainable alternatives. This work preliminarily fabricates starch- and gelatin-based composite films modified with natural fillers, and characterizes their basic physicochemical properties, dyeing performance, mechanical behavior, soil burial mass loss, and seed germination responses. On the basis of the tested baseline properties, tentative conceptual design schemes for eco-friendly toys were proposed. Notably, all experimental results in this work are preliminary and limited by insufficient experimental replicates. This study provides only basic experimental data and preliminary feasibility references for developing natural polymer composites for low-carbon consumer product scenarios.

## 1. Introduction

Bio-based composites are multiphase solid materials formed by combining one or more components derived from biomass through physical, chemical, or biological methods. These composites typically consist of a continuous matrix phase and dispersed reinforcement phases [1]. The reinforcement phase is embedded within the matrix to enhance the overall mechanical performance of the composite. It typically contributes strength, stiffness, or other functional characteristics, whereas the matrix provides continuous support, adhesion, and processability [2]. The matrix phase is the continuous material that forms the composite system [3]. The transition of bio-based composites from laboratory research to large-scale commercial applications is taking place, owing to increasing demand for sustainable materials in various sectors. The change is helping to fuel continued expansion in the global market. This trend is further illustrated by the increased use of these materials in high-value sectors such as automotive [4], construction [5], packaging [6], and medical applications [7].

Reinforcement phases commonly consist of natural fibers or other natural fillers (e.g., wood fibers, flax, cotton, animal hair, and natural clay) [8]. For example, Gioffrè et al. prepared hemp-based composites for masonry reinforcement by embedding commercial hemp ropes as reinforcement in different natural matrices. These composites showed a significant improvement in load-bearing capacity, which shows their effectiveness for engineering applications [9]. Figueroa-Velarde et al. used Mexican agave residue fiber as reinforcement, mixed with PLA at a specific proportion, and developed the composite by FDM [10]. The matrix can be a bio-based polymer (e.g., polylactic acid and polyhydroxyalkanoates) or a conventional polymer; bio-based matrices generally are derived from renewable biomass [11]. Starch and gelatin are natural polymers and typical matrix materials for bio-based composites [12]. For instance, Rivera-Muñoz et al. combined an aqueous solution of gelatin (matrix phase) and porous hydroxyapatite ceramic (inorganic phase) through an impregnation process. The obtained material showed good biocompatibility and mechanical compatibility, supporting its application for replacement of bone tissue or implants [13]. Kalateh-Seifari et al. prepared a composite film by solution casting, using a corn starch/chitosan blend as the matrix, starch nanocrystals as the reinforcement phase, and nettle oil nano-emulsion as the functional component. The material exhibited excellent mechanical properties and bioactivity, indicating its potential for food-packaging applications [14]. In summary, starch-based and gelatin-based composite materials have attracted considerable research and industrial interest because of their renewability, biodegradability, and good environmental compatibility. Accordingly, these two material systems were selected as representative examples for examining synthesis strategies and basic performance of bio-based composites.

Starch-based composites typically consist of starch and one or more other bio-based or synthetic materials [15]. Starch is a natural polysaccharide derived from plants. Combining starch with other materials (such as PLA, polyethylene, and polyurethane) can substantially improve the mechanical properties and water resistance of the resulting composites [16,17]. Such composites have a wide range of potential applications in agriculture, food packaging, biomedicine, and related fields [18]. Starch-based composites can generally be classified into two categories: fiber-reinforced starch-based composite materials and polymer-blended starch-based composite materials [19,20]. For fiber-reinforced composites, Vallejos et al. investigated fiber-reinforced composites using corn starch and cassava starch as matrix materials. Graded bagasse fiber separated in an ethanol–water system was added as the reinforcement phase, and the mixture was then pressed into its final shape. The results showed that adding sugarcane bagasse fiber significantly improved the composites’ mechanical properties and water resistance [21]. Le et al. developed an effective wheat-starch–hemp-fiber composite by optimizing the rheological properties of the starch binder. Their study indicated that the material achieved the best combination of lightness and deformability when the binder had high viscosity and low surface tension. This combination made the composite suitable for building infill applications [22]. For polymer-blended composites, Liang et al. used carboxymethyl starch and polyvinyl alcohol as matrix components, cross-linked the metal-organic framework HKUST-1 within the matrix, and loaded curcumin to prepare a bio-based fruit preservation film with antibacterial properties under high-humidity conditions [23]. Shuprajhaa et al. prepared banana-starch-based composite films by casting, using glycerol as the plasticizer and blending polyvinyl alcohol and carboxymethyl cellulose at different proportions. The effects of the two additives on film properties, particularly degradation behavior, were then compared [24].

Gelatin-based composites are multiphase systems formed by blending, cross-linking, or chemically modifying gelatin (a natural protein polymer obtained from the hydrolysis of animal collagen) with other natural or synthetic polymers, inorganic fillers, or nanomaterials, with gelatin serving as the matrix or a key component [25]. The properties of gelatin-based composites depend strongly on composition and design strategy. For example, gelatin undergoes a colloidal-gel transition between 35 and 40 °C. By introducing photo-initiators or applying chemical modification, gelatin can be endowed with photocuring or temperature-sensitive gelation capabilities. These properties make gelatin-based materials well suited to bio-3D printing, enabling precise fabrication of cell scaffolds with complex three-dimensional structures and supporting applications in tissue engineering [26]. Lehman et al. showed that introducing 10 wt% Moringa oleifera pod fibers into a gelatin matrix resulted in a composite with optimal overall performance: both tensile strength and Young’s modulus reached their maximum values, and water vapor barrier properties were significantly improved. This simultaneous improvement was attributed to efficient stress transfer at the fiber–matrix interface and a denser microstructure. These findings provide guidance for the design of environmentally friendly, high-performance packaging materials [27].

Composites based on starch and gelatin could also have good biodegradability, an important feature to improve sustainability and reduce environmental pollution. For starch-based systems, researchers have fabricated starch-based composite nanofiber membranes (SNFs/TA/Fe^3+^) for active food packaging. The addition of tannic acid (TA) and ferric ions (Fe^3+^) enhanced the mechanical strength and the antioxidant activity of these membranes [28]. Corn-starch-based composites reinforced with Jaipur palm fiber have good thermal stability and mechanical properties and can be used in automotive and electronics applications [29]. Thermal stability and mechanical strength can be greatly improved by the combination of maize starch with nanomaterials such as microcrystalline cellulose. Such properties render the material suitable for application as a thermal insulation layer or structural component of aerospace vehicles [30]. The bio-based composite (matrix: corn starch; reinforcement: jute fiber waste) exhibited good thermal conductivity, tensile strength, hardness, and water resistance. These properties make it suitable for thermal insulation in construction and industrial applications [31]. Composite films based on gelatin (EG) extracted from leather waste and polylactic acid (PLA) have been prepared for gelatin-based systems. The optimization of the EG ratio, flexibility, thermal processability, and biodegradability can be used to balance performance and environmental considerations in food-packaging applications [32]. Composite materials based on gelatin are prepared by mixing gelatin with polysaccharides, nanomaterials, and other additives. These materials can provide good bio-absorbability and biosafety. They degrade into harmless byproducts such as amino acids and peptides. It is possible to tune the degradation rate through the manipulation of various parameters such as cross-linking and porosity. These features favor biomedical applications of the composites, such as tissue engineering scaffolds and pharmaceutical delivery systems [33]. The composite of gelatin, agarose, zeolite, and pomegranate peel extract shows good mechanical stability and controlled porosity. It supports cell compatibility, controlled drug release, and swelling regulation. These properties support potential application in skin regeneration and repair in tissue engineering and skincare applications [34]. Overall, starch- and gelatin-based composites are being explored in a variety of application areas, as part of wider efforts to develop more environmentally friendly and sustainable materials.

Previous studies have predominantly focused on optimizing specific preparation processes or improving individual material properties. Although bio-based composites have been considered for conceptual toy applications, dedicated comparative explorations of both starch-based and gelatin-based composites targeting potential eco-friendly toy scenarios remain scarce. Against this background, the present work performs a preliminary laboratory-scale investigation of two independent natural polymer composite systems. A set of basic mechanical, coloring, soil burial mass loss, and seed germination tests was performed to preliminarily assess the potential of the two material systems. On this basis, conceptual toy design prospects were further discussed. This study aims to provide fundamental experimental data and preliminary feasibility exploration for the development of low-cost bio-based materials intended for potential eco-friendly toy scenarios. Despite increasing diversification and technological integration in toy design, conventional plastics such as polyethylene and polypropylene still dominate material selection [35]. Although these materials are inexpensive and readily processed, they also raise environmental and safety concerns. For example, harmful substances such as lead and cadmium have been reported in some plastic toys [36]. These substances can be ingested, contacted, or abraded by a child and may be hazardous to health [37]. Several research efforts have explored sustainable toy designs with natural materials [38,39] and shown edible and biocompatible materials for use in infant care [40,41]. However, the focus on single-material systems and the high cost of sourcing raw materials have hindered their widespread use. This study therefore further investigates the incorporation of starch-based and gelatin-based composites into conceptual toy design. The method also considers the possible use of eco-friendly materials in consumer goods. One potential advantage of these bio-based materials may be compatibility with product design oriented toward sustainability, although suitability for children’s products requires dedicated safety validation. Their natural textures may also offer opportunities for tactile design and environmental education. Moreover, their biomass-based composition offers a chance to decrease the reliance on traditional plastics. They also have potential for cost-efficient processing as their laboratory preparation is relatively simple, but scalability needs to be further validated. Combined, these features provide a basis for exploring more circular material strategies in toy design.

The remaining sections of this document are organized as follows. Section 2 describes the preparation of the starch- and gelatin-based composites and the methods used for their assessment. Section 3 describes the results of dyeing tests, mechanical characterization, mass loss tests in soil burial, seed germination tests, and conceptual toy design. Section 4 discusses the findings and limitations of the study, and Section 5 concludes the study.

## 2. Materials and Methods

### 2.1. Overall Approach

**Figure 1:**
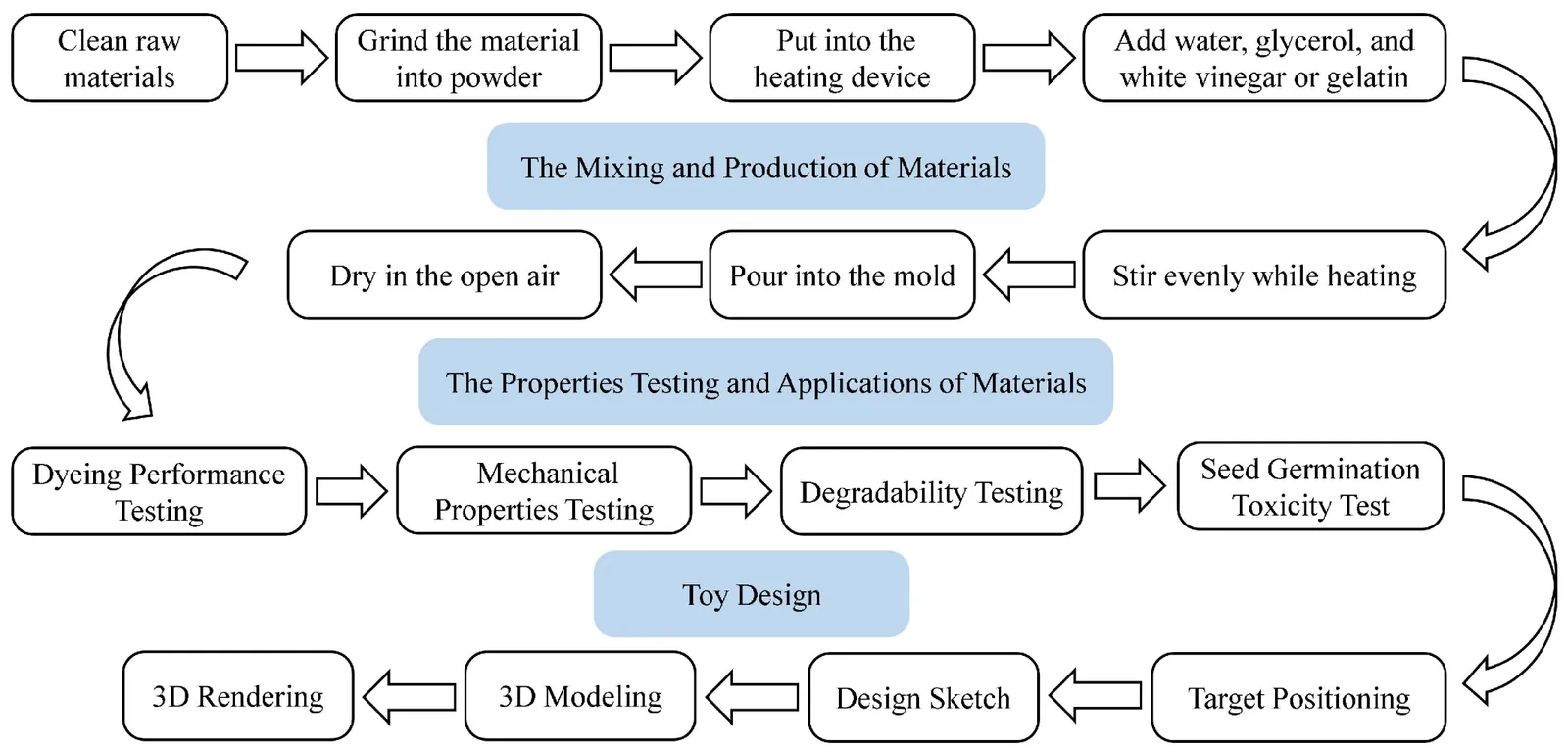
Overall concept diagram.

Considerable research has examined preparation pathways for individual composite systems [42,43,44,45,46,47,48,49,50]. Nevertheless, few of these studies integrate multiple basic property characterizations to explore the potential application of starch- and gelatin-based composites in conceptual eco-friendly toy designs. Using starch- and gelatin-based composites incorporating natural fillers, this study describes the fabrication procedure for the target composite systems, performs a set of basic laboratory-scale characterization tests, and conceptually examines their potential relevance to toy design. The overall experimental approach is shown in Figure 1. First, corn starch and orange-peel powder were selected as raw materials, mainly based on the following considerations: Corn starch is widely available, inexpensive, and has favorable film-forming ability and biodegradability, making it a representative polysaccharide matrix for bio-based composites [51]. Large quantities of orange-peel waste are generated each year globally, and improper disposal can impose an environmental burden. At the same time, using orange-peel powder may improve resource utilization by valorizing an agricultural byproduct [52]. Accordingly, corn starch and orange-peel powder were incorporated into the respective starch- and gelatin-based formulations.

Regarding the specific preparation routes, the preparation of orange-peel-gelatin-based composite materials comprised three main steps. First, orange peel was washed to remove surface impurities, then dried and ground into a fine powder. Next, orange-peel powder, gelatin, glycerin, and water were mixed at the specified proportions and heated under stirring to form a homogeneous mixture. Finally, the mixture was cast into a mold and air-dried to obtain the final product. Corn-starch-based composites were prepared using a similar procedure. Corn starch, white vinegar, glycerin, and water were mixed at specified proportions, followed by heating, stirring, molding, and air-drying. Although the two systems differ in matrix composition and raw-material sources, their core preparation steps are similar, indicating a common processing framework. Accordingly, the common preparation route can be summarized as raw material pretreatment, controlled ingredient mixing, thermal processing and gelatinization/plasticization, molding, and natural air-drying to constant weight. Starch and gelatin were used as model matrix systems to examine the feasibility of this preparation route. After material formation, dyeing performance, mechanical properties (including tensile strength and flexibility), soil burial mass loss behavior, and seed germination responses were evaluated. Based on these evaluations, two conceptual toy designs were proposed to explore potential application scenarios for the materials.

### 2.2. Material Preparation

**Figure 2:**
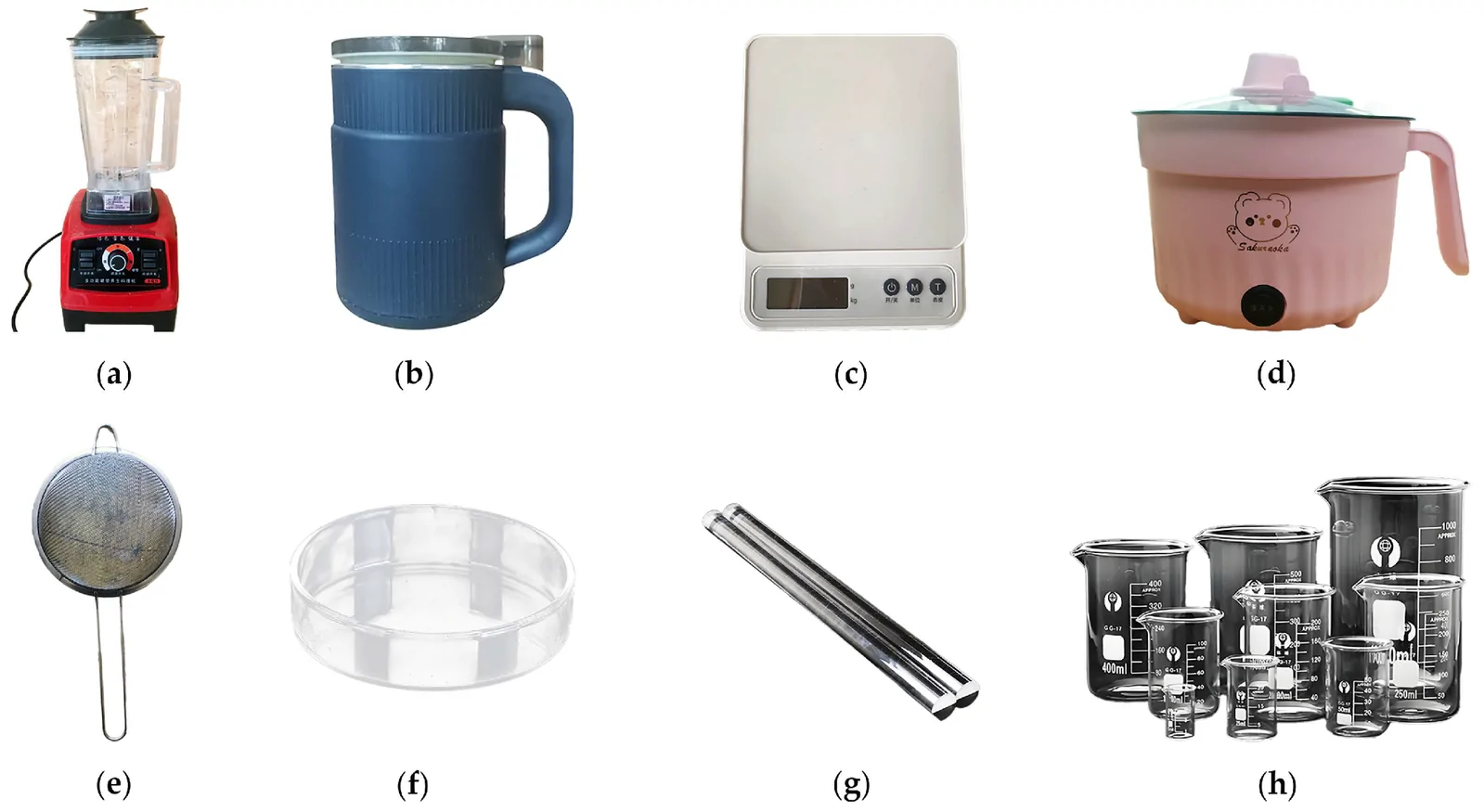
Equipment used for material preparation. (**a**) “Baijiamei” high-speed blender; (**b**) “Xinfei” food processor; (**c**) “Junsheng Huiyi” electronic scale; (**d**) “Fengbin” electric cooker; (**e**) “Qiaoxing” stainless-steel handheld flour sifter, 14 cm; (**f**) “Dippo” disposable sterile plastic petri dish, 90 mm; (**g**) “Baibo” glass rod; (**h**) “Baibo” glass beakers.

**Figure 3:**
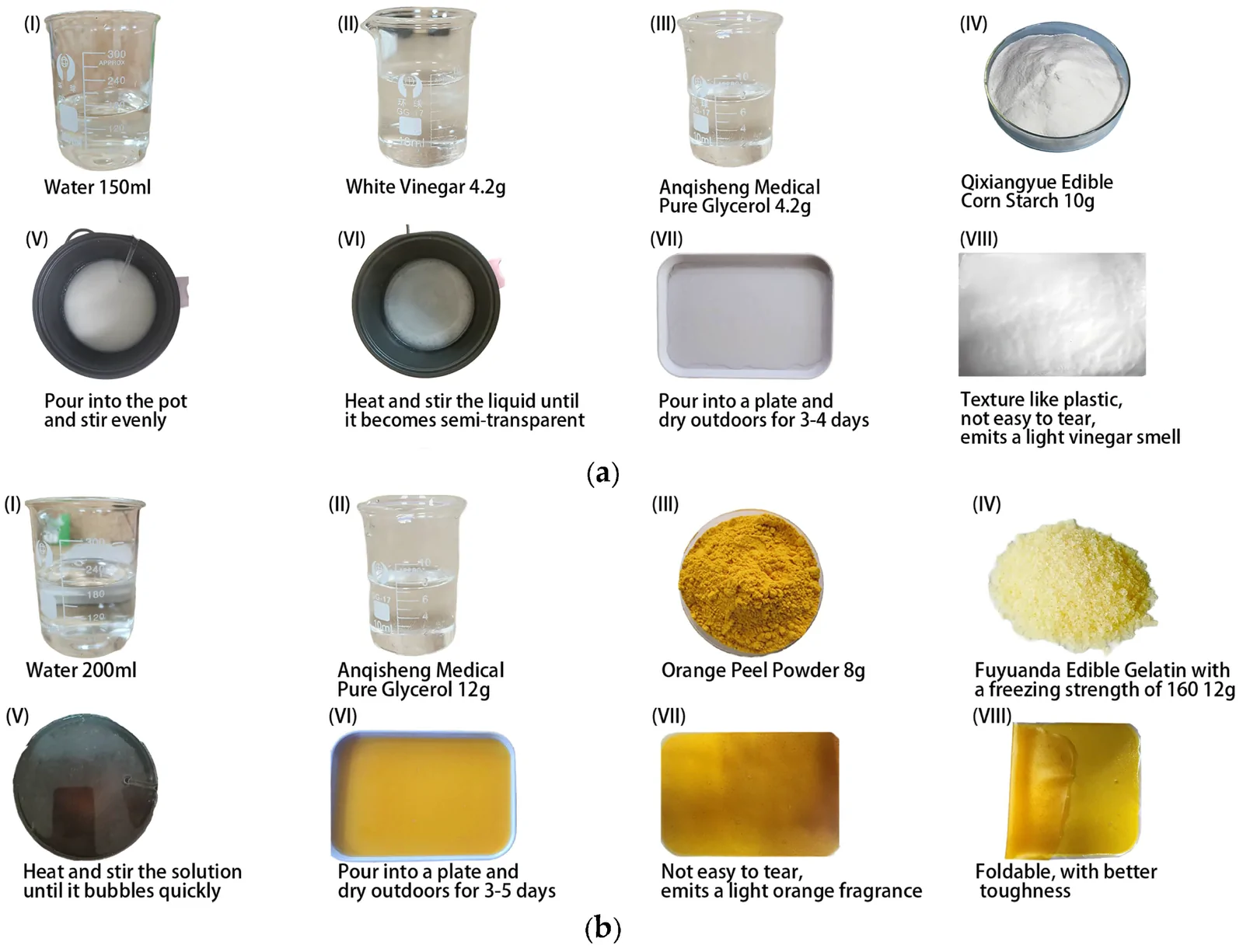
Material preparation procedures. (**a**): (**I**–**VIII**) Preparation process for corn-starch-based composites; (**b**) (**I**–**VIII**) preparation process for orange-peel-gelatin-based composites.

The equipment used for material fabrication is shown in Figure 2. Corn-starch-based composites were prepared as follows (Figure 3a): First, 10 g of corn starch (Qixiangyue, Shijiazhuang, China) and 150 mL of tap water were weighed. The mixture was transferred to an electric cooker (Fengbin, Guangdong, China) and stirred thoroughly with a glass rod (Baibo, Yangzhou, China) to obtain a homogeneous suspension. Subsequently, 4.2 g of glycerol (Anqisheng, Shanghai, China) and 4.2 g of acetic acid (Hengshun, Zhenjiang, China) were then added sequentially. The mixture was then heated at 60 °C under stirring for 2 min. Heating was stopped immediately when the mixture transformed into a translucent gel. After heating, surface foam was removed, and the gel was cast into a mold to obtain a thickness of 1 mm. The cast sample was then placed on a well-ventilated, shaded indoor balcony and air-dried at room temperature (25 ± 2 °C) for 6 days. The final moisture content of the demolded starch composite specimens was determined by the gravimetric method. Approximately 2 g of samples (small fragments were cut from the composite sheet) were oven-dried at 105 °C to constant mass, cooled to ambient temperature inside a desiccator, and weighed in triplicate. The measured final moisture content was 6.2 ± 0.4 wt%. All physicochemical characterizations were performed on samples at this moisture state. All other formulation groups were air-dried and stored under the same ambient conditions (25 ± 2 °C, ventilated dark environment) to achieve equilibrium moisture levels comparable to those of the baseline samples before characterization. This formulation was used as the baseline.

Orange-peel-gelatin-based composites were prepared as follows (Figure 3b): First, the orange peels were placed in a well-ventilated, dark indoor environment and air-dried until constant weight was achieved. The dried peels were then ground using a food processor (Xinfei, Jinhua, China) and passed through a 30-mesh standard sieve (Qiaoxing, Chaozhou, China) to obtain orange-peel powder with particle sizes below approximately 600 μm. Due to the exploratory nature of this preliminary study, full particle size distribution measurement was not performed. Next, 8 g of orange-peel powder, 12 g of gelatin (Fuyuanda, Zhoukou, China), 12 g of glycerol, and 200 mL of tap water were weighed. These ingredients were added to an electric cooker and stirred thoroughly with a glass rod to obtain a uniform dispersion. The mixture was heated at 60 °C under stirring for 2 min, after which heating was immediately stopped. After heating, surface bubbles were removed from the mixture, and the mixture was cast into a mold (with a thickness of 1 mm). Finally, the mold was placed on a well-ventilated, shaded indoor balcony and air-dried at room temperature (25 ± 2 °C) for 8 days. The sample was then demolded. The final moisture content of demolded orange-peel-gelatin-based composites was tested following the identical gravimetric procedure described above. The specimens exhibited a final moisture content of 7.8 ± 0.4 wt%, and all subsequent physicochemical tests were conducted under this moisture condition. All other formulation groups were air-dried and stored under the same ambient conditions to achieve equilibrium moisture levels comparable to those of the baseline samples before characterization. This formulation was used as the baseline.

### 2.3. Experimental Methods

#### 2.3.1. Dyeing Test

Plant juices from blueberry, tomato, dragon fruit, spinach, purple cabbage, and tangerine were used as natural pigments for dyeing. This approach was used to assess the compatibility of the two composites with plant-derived pigments. Fresh plant materials were pressed and filtered through filter paper to remove solid residues, yielding clarified pigment liquids. For corn-starch-based composites, 150 mL of tap water, 10 g of corn starch, 4.2 g of glycerol, and 4.2 g of acetic acid were weighed according to the baseline formulation. Subsequently, each formulation group was mixed with 10 g of prepared plant pigment liquid. The raw materials were directly added into an electric cooker and heated at a temperature of 60 °C with constant stirring for 2 min until gelatinization (the mixture turned uniformly translucent). The gelatinized mixture was cast in molds with controlled thicknesses of 1 and 5 mm for color comparison and dried for 6 days under ventilated, light-shielded constant-temperature (25 ± 2 °C) conditions.

For orange-peel-gelatin-based composites, the dosage of orange-peel powder was fixed at 3 g to minimize the intrinsic color interference of orange-peel powder on the dyeing results. The base formulation consisted of 200 mL tap water, 12 g glycerol, 12 g gelatin, and 12 g plant pigment liquid. Raw materials were heated in an electric cooker at 60 °C with constant stirring for 2 min and then cast into molds of 1 mm and 5 mm thicknesses. Samples were dried for 8 days in the same ventilated, light-shielded condition (25 ± 2 °C). Subsequently, the macroscopic visual inspection was employed for the preliminary qualitative comparison of the coloring effects of different sample groups after drying. Remarkably, there were no independent sample replicates prepared for this series of dyeing trials.

#### 2.3.2. Mechanical Testing

**Figure 4:**
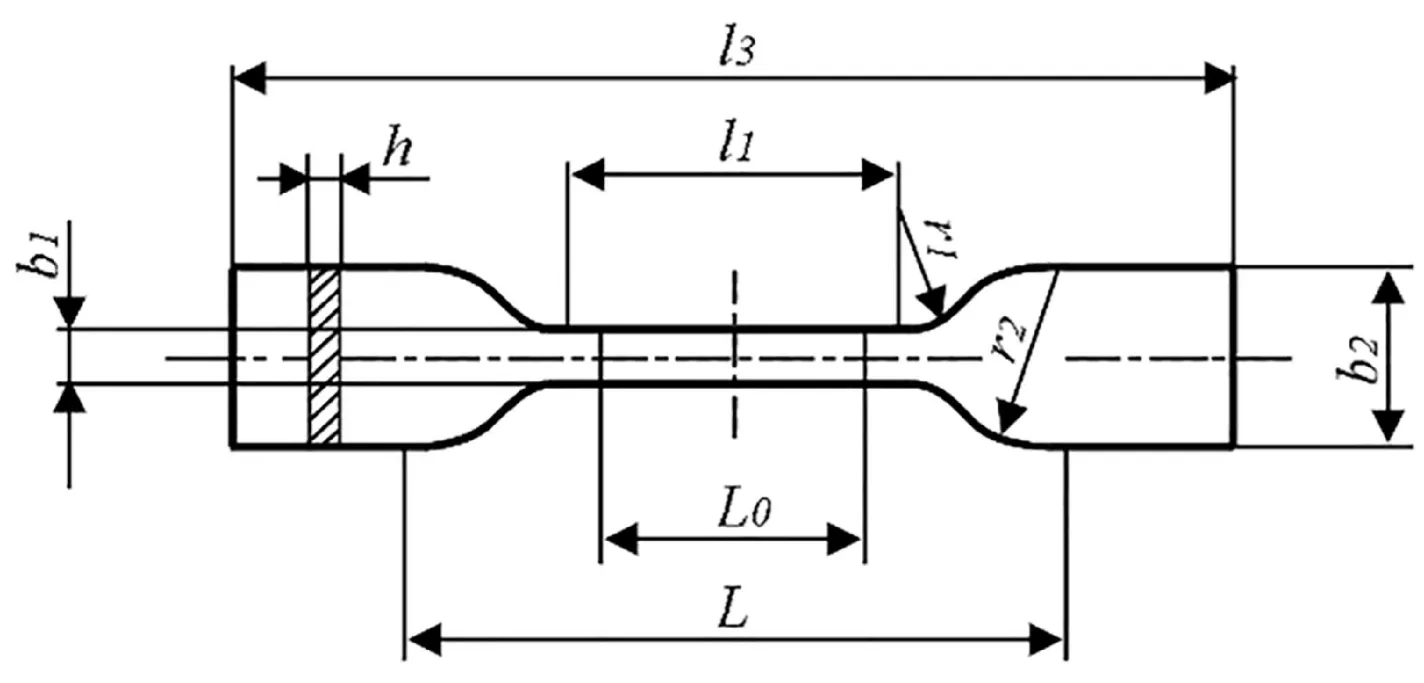
Specimen geometry based on the Type 5A configuration in GB/T 1040.2-2022.

**Figure 5:**
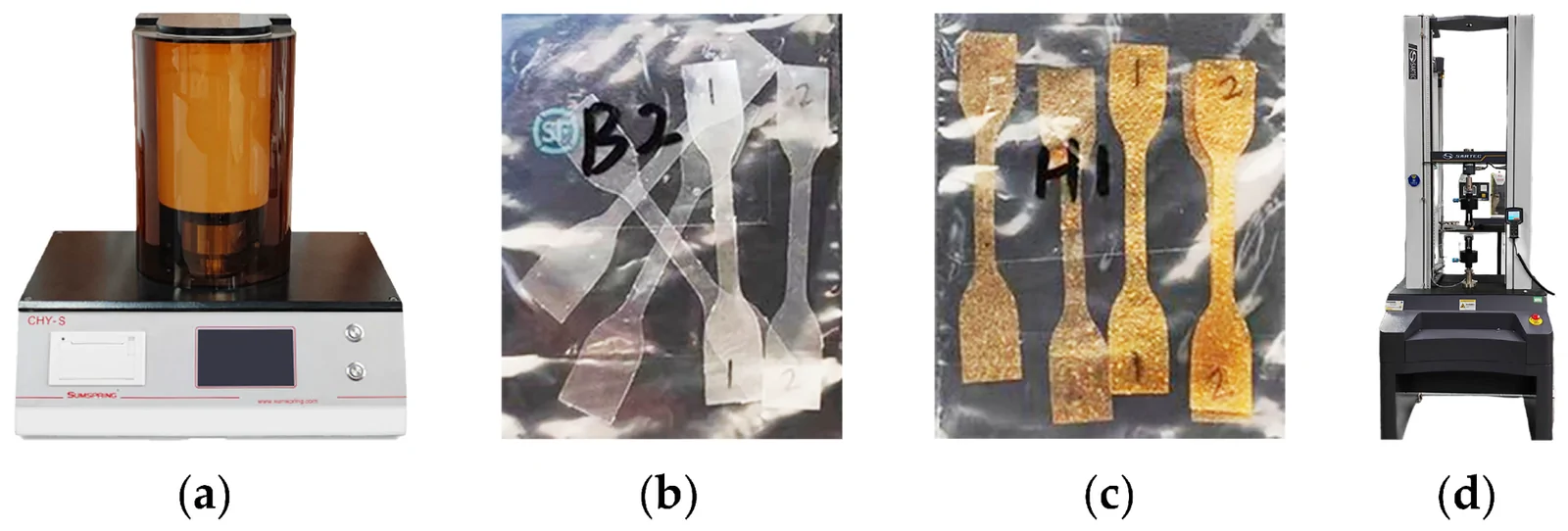
Equipment and specimens used for mechanical testing. (**a**) “SUMSPRING” CHY-HS thickness gauge; (**b**) corn-starch-based composite specimen; (**c**) orange-peel-gelatin-based composite specimen; (**d**) “Sungshu” SS-8600-1KN universal tensile testing machine.

Uniaxial tensile tests were performed to evaluate the mechanical properties of corn-starch-based and orange-peel-gelatin-based composite films. All specimens were fabricated according to GB/T 1040.2-2022 (Type 5A) [53], with detailed dimensions listed in Table 1 and the overall geometry illustrated in Figure 4. Specimen thickness was accurately measured using a CHY-HS thickness gauge (SUMSPRING, Jinan, China) prior to testing. All tensile measurements were conducted using an SS-8600-1KN universal testing machine (Sungshu, Guangdong, China) at a constant crosshead speed of 5 mm/min. Real-time force–displacement data and fracture morphologies were recorded throughout the tests. For the two baseline formulations, two parallel specimens (n = 2) were tested per material. For modified formulations with varied component ratios, mechanical tests were performed without independent sample replication, and repeated technical measurements were applied to minimize experimental error (Figure 5).

Force and displacement data were converted to engineering stress and strain. Engineering stress was calculated as follows:

(1) $$\sigma_{e}=\frac{F}{A_{0}}$$

where *σ_e_* is the engineering stress, *F* is the applied force, and *A*_0_ is the initial cross-sectional area; for rectangular specimens, *A*_0_ = *w*_0_ × *t*_0_, where *w*_0_ is the initial width, and *t*_0_ is the initial thickness.

Engineering strain was calculated as follows:

(2) $$\varepsilon_{e}=\frac{\Delta L}{L_{0}}=\frac{L-L_{0}}{L_{0}}$$

where *ε_e_* is the engineering strain, ∆*L* is the change in length, *L*_0_ is the initial gauge length, and *L* is the current length.

Young’s modulus (*E*) was determined from the slope of the initial linear region of the engineering stress–strain curve. It was calculated as follows:

(3) $$E=\frac{\sigma_{e}}{\varepsilon_{e}}$$

All data were processed in MATLAB (R2024a, MathWorks, Natick, MA, USA) to generate force–displacement and engineering stress–strain curves.

#### 2.3.3. Soil Burial Test

**Figure 6:**
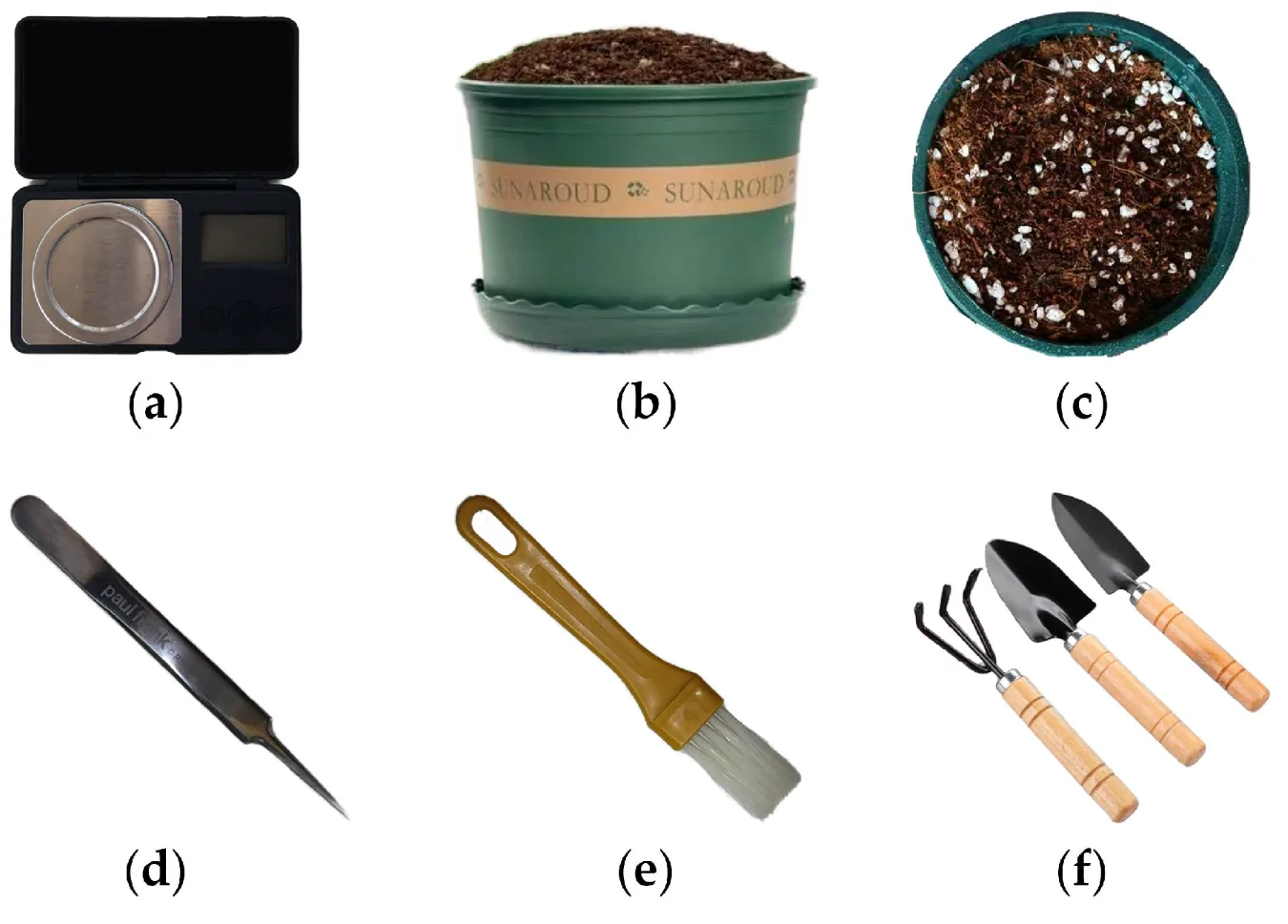
Equipment used for the soil burial mass loss test. (**a**) “Coolbee” high-precision jewelry electronic scale (0.001 g–10 g); (**b**) “Youthful Seasons Gardening” medium gallon pot; (**c**) “Youthful Seasons Gardening” nutrient soil; (**d**) “Paul Frank” pointed-nose tweezers; (**e**) “Angel Baby” no. 8 BBQ oil brush; (**f**) “Cangqiang” small gardening shovel 3-piece set.

The soil burial method was used to evaluate the mass variation behavior of corn-starch-based and orange-peel-gelatin-based composites. The experimental setup is shown in Figure 6. Samples were first cut to 2 × 2 cm, and their initial mass was recorded. The initial mass of corn-starch-based and orange-peel-gelatin-based specimens was 0.106 g and 0.122 g, respectively. A preliminary trial was conducted before the formal test to observe mass change trends and determine an appropriate sampling interval according to the actual rate of mass variation of each material. The two materials were then buried in separate containers containing equal amounts of homogeneous nutrient soil (coconut coir: perlite: peat moss) (Youthful Seasons Gardening, Heze, China) and placed under the same environmental conditions. The nutrient soil possessed pH 5.8–6.5, organic matter content ≥ 45%, and total nitrogen content 0.5–0.8%. No additional chemical fertilizer or bactericide was added. All containers were placed under consistent semi-open balcony conditions, with ambient temperature fluctuating from 22 °C to 30 °C and protection from direct sunlight. Soil moisture was maintained by regular watering following the same regime used in the seed germination assay.

This soil burial mass loss test was carried out as a single experimental trial without independent parallel replicates. Sampling was performed every 2 h throughout the incubation period. During sampling, soil was gently loosened with a small shovel, and specimens were carefully retrieved using tweezers. Adhered soil particles were softly brushed away from sample surfaces, followed by natural air-drying in a well-ventilated environment until constant weight was reached. Each dried specimen was weighed three times using an electronic balance (Coolbee, Jinhua, China), and the average mass value was recorded. These repeated weighings served as technical replicates to reduce weighing error for the same specimen, rather than independent sample replicates.

The mass loss rate was calculated according to the following formula:

(4) $$\mathrm{Mass}\;\mathrm{loss}\;\mathrm{rate}\;\left(\%\right)=\frac{m_{0}-m_{t}}{m_{0}}\times 100\%$$

In the formula, *m*_0_ is the initial mass, and *m_t_* represents the specimen mass at the burial time *t*.

Notably, the calculated mass loss rate only reflects the macroscopic mass variation of samples after soil burial, and cannot confirm microbial biodegradation. After weighing, each specimen was returned to its original burial position to preserve the local soil microenvironment.

#### 2.3.4. Seed Germination Assay

**Figure 7:**
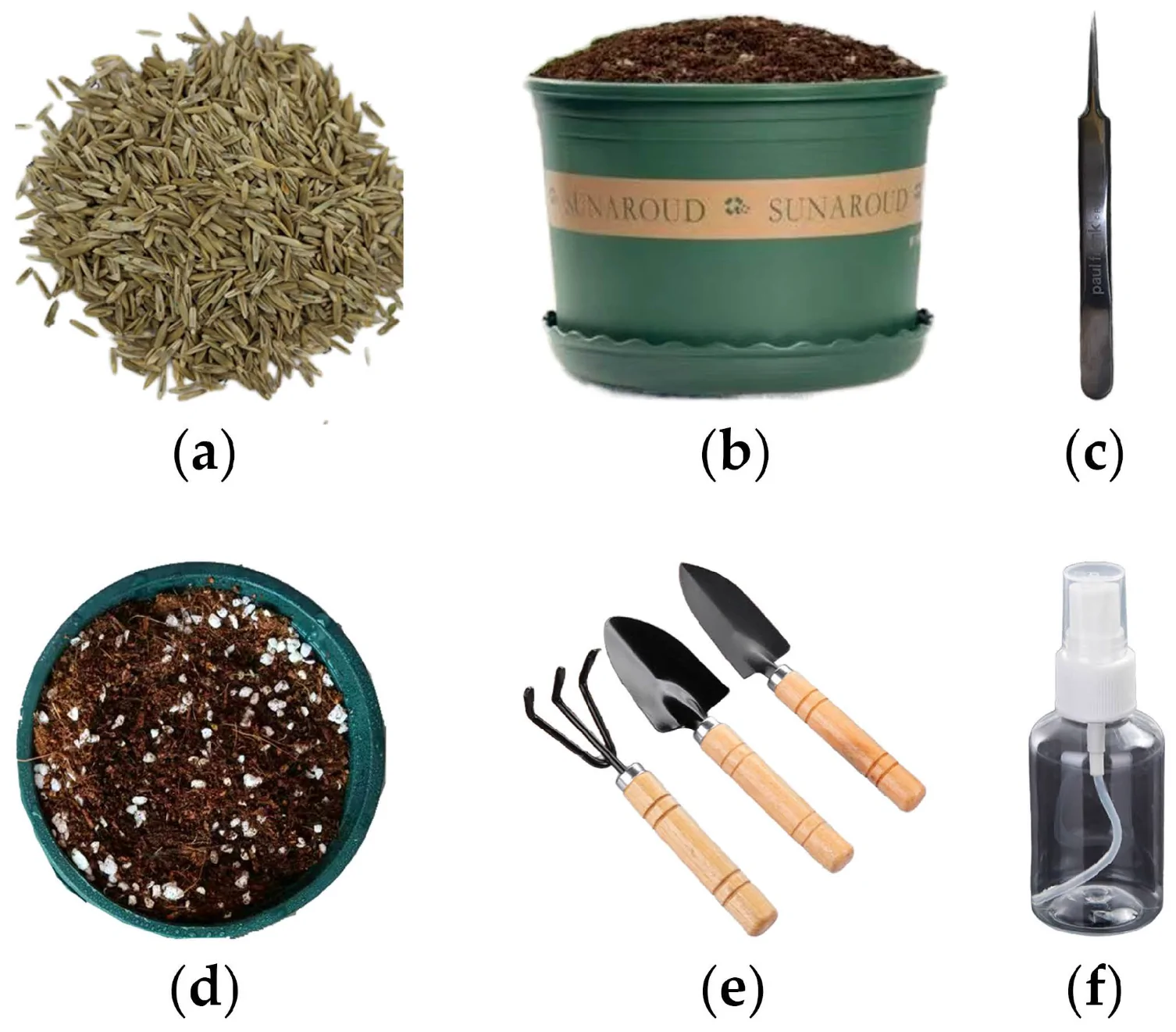
Equipment used for the seed germination test. (**a**) Ryegrass seeds; (**b**) “Youthful Seasons Gardening” medium gallon pot; (**c**) “Paul Frank” pointed-nose tweezers; (**d**) “Youthful Seasons Gardening” nutrient soil; (**e**) “Cangqiang” small gardening shovel 3-piece set; (**f**) “Mars Farm” mini-sprayer (10 mL).

**Figure 8:**
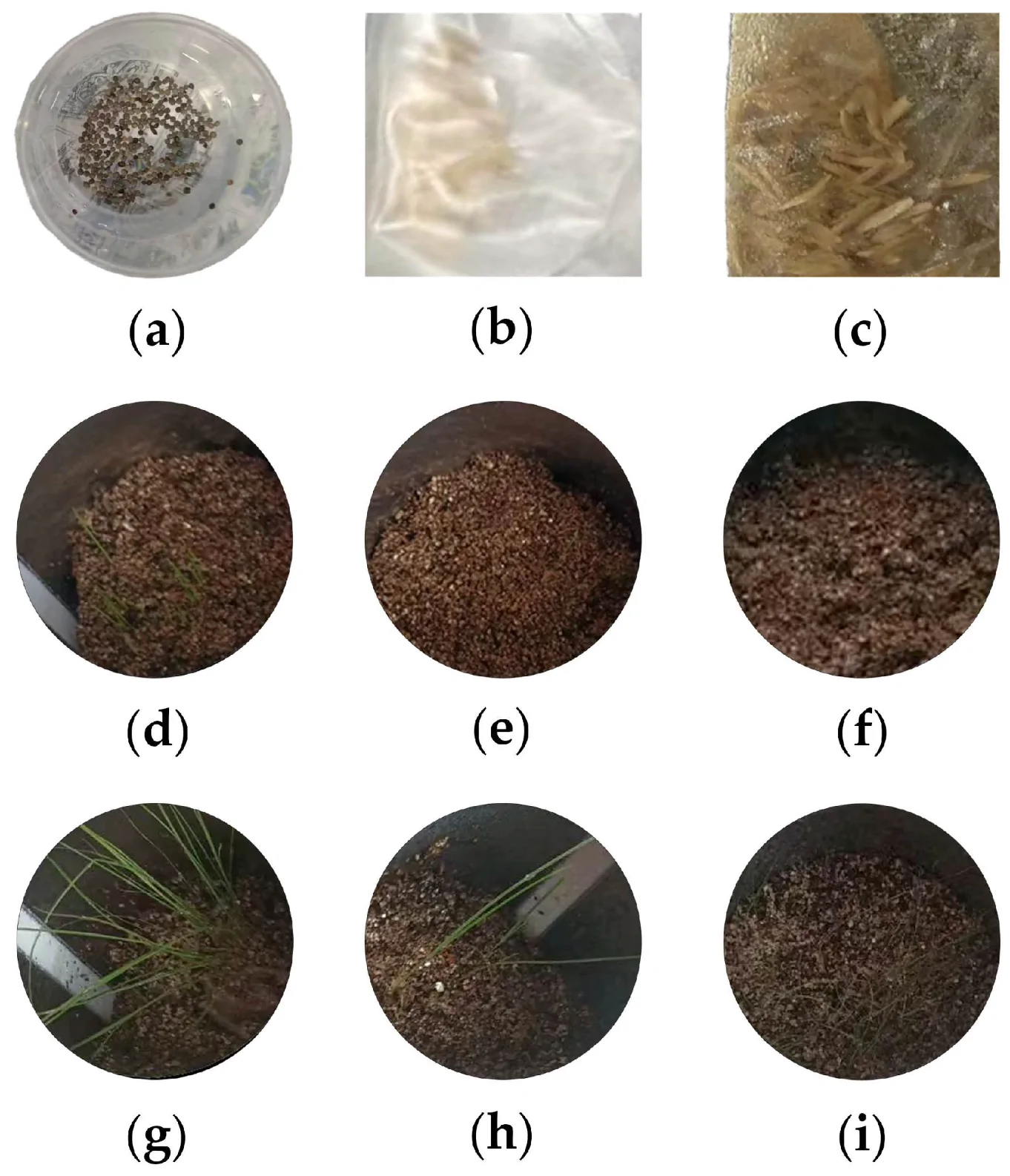
Procedure for the seed germination test. (**a**) Seeds soaked in warm water; (**b**) seeds coated with corn-starch-based composite film; (**c**) seeds coated with orange-peel-gelatin-based composite film; (**d**) early germination of naked seeds; (**e**) early germination of seeds coated with corn-starch-based composite film; (**f**) early germination of seeds coated with orange-peel-gelatin-based composite film; (**g**) late germination of naked seeds; (**h**) late germination of seeds coated with corn-starch-based composite film; (**i**) late germination of seeds coated with orange-peel-gelatin-based composite film.

Plants are sensitive to potentially harmful substances in the environment, including heavy metals and small-molecule organic compounds released from materials. If a material or its degradation products are phytotoxic, they may inhibit germination by interfering with seed-enzyme activity or damaging seedling cell membranes, thereby impairing photosynthesis, respiration, and subsequent root or seedling growth [54]. This trial tentatively compared the germination behavior of film-wrapped seeds with uncovered control seeds to monitor differences in germination kinetics and final germination proportions. Germination progression and final germination percentages were recorded to document seed performance under the tested conditions, yielding preliminary observational data. The apparatus used in the experiment is shown in Figure 7, and the technique is shown in Figure 8. This germination assay was an exploratory single-batch investigation with no independent biological replicates. Each cluster is made up of 35 seeds of ryegrass. So, in this study a total of 105 seeds is used. The ryegrass seeds (initially 105) were soaked in water at 28 °C for 1.5 h. The seeds, after soaking, were carefully placed on a sterile filter paper using a pair of tweezers, and the excess water was removed. Then, 35 seeds were encapsulated in composite films of corn starch and orange-peel gelatin, with a thickness of 0.20± 0.02 mm, prepared according to the standard formulations. The seeds were placed flat on the film and completely covered without creasing them too much. The film edges were moistened with water to improve adhesion and maintain close contact with the seeds without visible gaps. A control was established with “corn-starch-based composite film coating”, “orange-peel-gelatin-based composite film coating”, and “naked seeds” (the control group also contained 35 uncoated seeds). Each flowerpot was filled with an equal amount of the same nutrient soil and placed at the same shaded balcony location away from direct sunlight. Seeds were sown at a uniform depth of 1.5 cm. An initial 30 mL of water was applied to each pot; 30 mL water was replenished daily at 9:00 a. m. to maintain consistent soil moisture. Seed germination was monitored and recorded daily to calculate germination rates. This provides preliminary data to evaluate the potential effects of the materials on seed germination.

## 3. Results

### 3.1. Dyeing Performance

**Figure 9:**
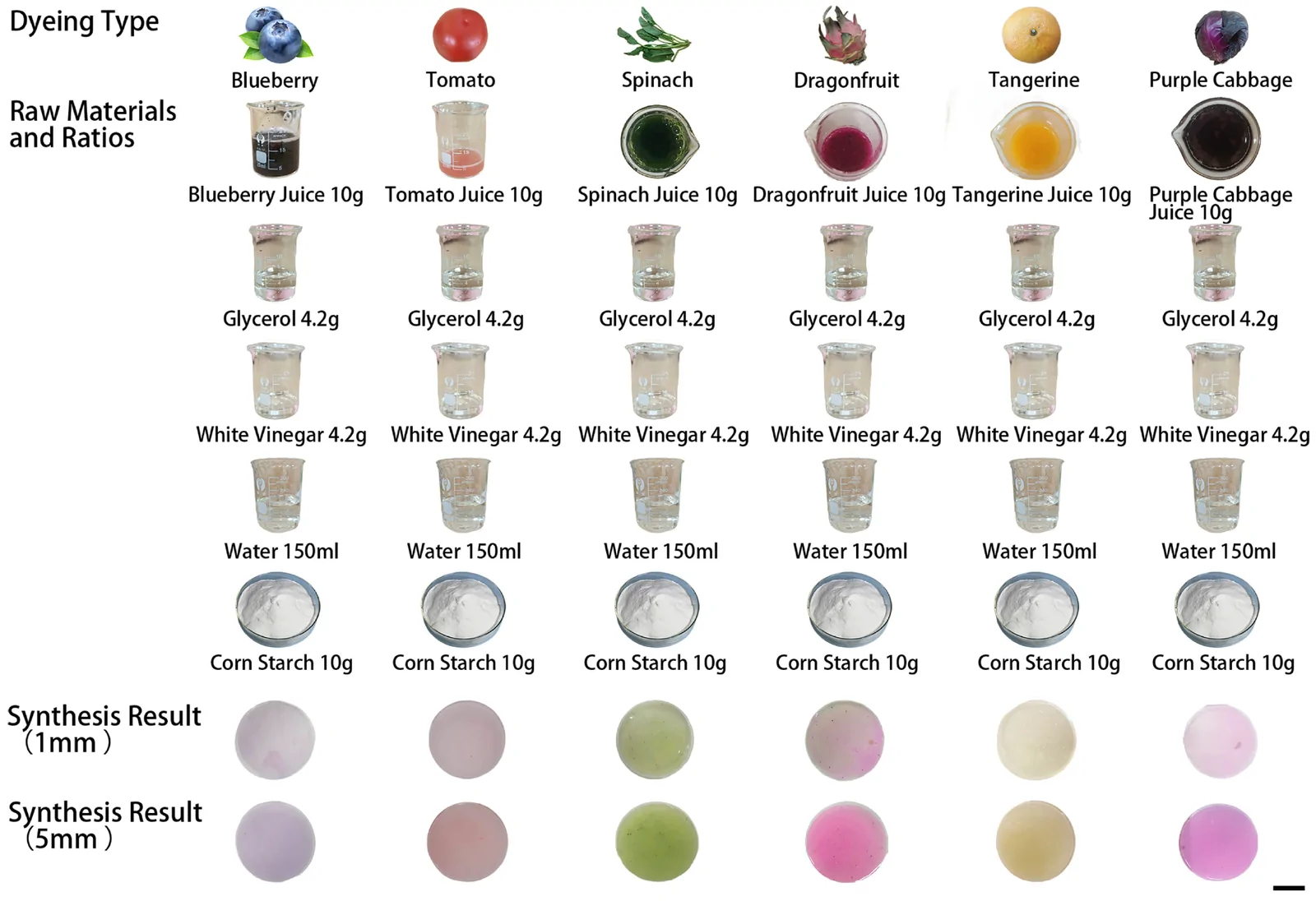
Preparation process for plant-dyed corn-starch-based composites. Scale bar: 4 mm.

This section presents the dyeing results for corn-starch-based and orange-peel-gelatin-based composite materials. These dyeing tests serve as a preliminary qualitative feasibility exploration, with the primary goal of verifying whether plant-derived pigments can impart color to biocomposite samples. Corn-starch-based composites were dyed by incorporating plant juices into fixed formulations of corn starch, water, glycerol, and white vinegar. The results are shown in Figure 9. The blueberry-dyed starch composite exhibited a blue-purple color. This coloration is attributable to anthocyanins in blueberry juice. Anthocyanins are water-soluble pigments widely distributed in plants. Anthocyanins can display colors ranging from red to blue depending on pH [55]. Tomato juice produced a reddish-orange hue. This coloration is associated with the high lycopene and carotenoid contents of tomato juice. According to Heriyanto et al., in corn-starch-based composites containing lycopene, both the a value (red) and the b value (yellow) increased with increasing corn-starch concentration. This trend is consistent with an orange to orange-red appearance [56]. Spinach, dragon fruit, orange, and purple cabbage juices produced yellow-green, pink, light yellow, and purple colors, respectively. These colors are associated with chlorophyll (spinach juice), betalain (dragon fruit juice), carotenoids (orange juice), and anthocyanins (purple cabbage juice). The color intensity was slightly different for the 1 mm and 5 mm specimens. The 1 mm samples appeared more luminous, while the 5 mm samples had a darker and more intense coloration. This observation may suggest potential differences in pigment distribution or concentration within the material. It is hypothesized that thicker specimens may contain more pigment, leading to the visually deeper color.

**Figure 10:**
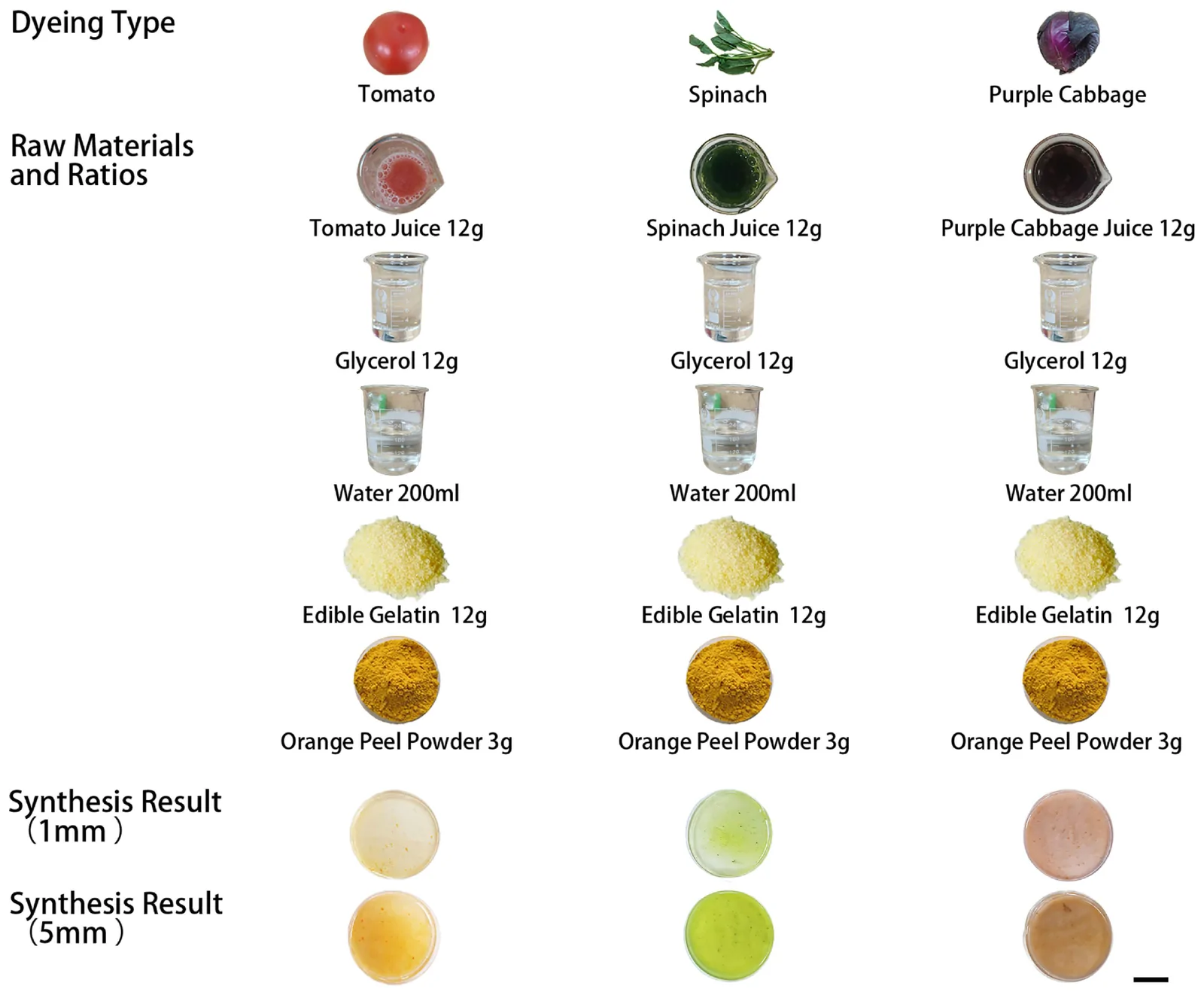
Preparation process for plant-dyed orange-peel-gelatin-based composites. Scale bar: 4 mm.

Composites based on gelatin obtained from orange peel were colored by heating the plant juice with defined ratios of glycerol, water, gelatin, and orange-peel powder. The results are presented in Figure 10. The gelatin composite dyed with tomato displayed an orange-yellow color. This color may be due to naringenin chalcone in the tomatoes, while gelatin may provide a more uniform distribution of pigment [57,58]. The spinach juice was yellowish green. This composite hue may be due to the different light-absorption properties of chlorophyll and carotenoids [59]. The juice of the purple cabbage was brown. Orange peel and purple cabbage juice are rich in oxidizable phenolic compounds including flavonoids and phenolic acids. These compounds may polymerize oxidatively during heating or storage to give brown products [60]. As in the starch-based system, the 1 and 5 mm specimens showed slight differences in apparent color. Overall, plant-derived pigments successfully imparted color to the composites. The tested pigments produced hues spanning red, orange, yellow, green, and purple. Notably, the present dyeing evaluation is limited to qualitative visual observation without quantitative color calibration (CIE L*a*b*) or assessments of color fastness, UV stability, wet-rub resistance, antimicrobial performance, and humidity stability. Therefore, the obtained coloring results remain preliminary and cannot support practical product-level performance verification for child-contact applications.

### 3.2. Mechanical Performance

#### 3.2.1. Basic Mechanical Properties

**Figure 11:**
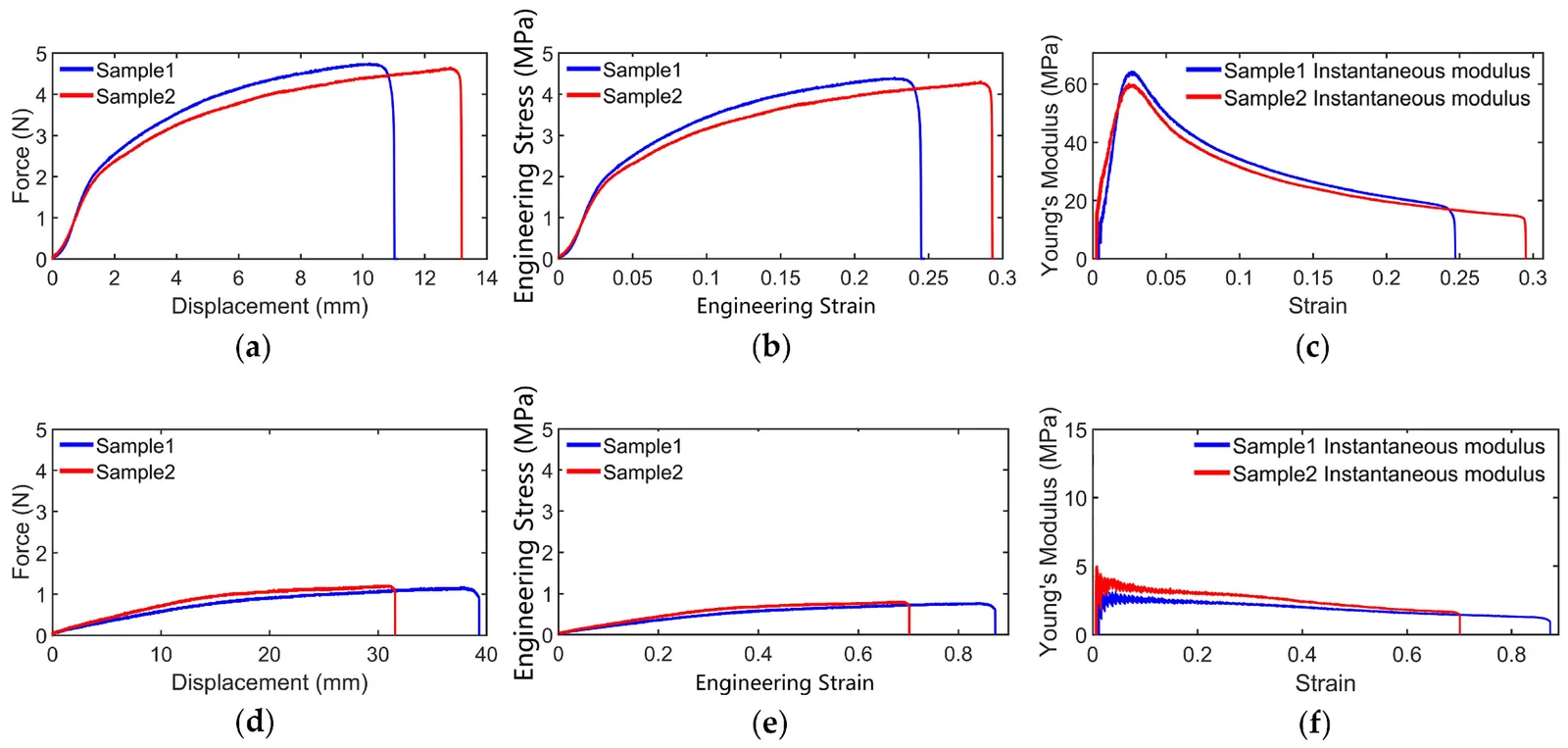
Mechanical responses of corn-starch-based composites and orange-peel-gelatin-based composites. (**a**) Force–displacement curves of the corn-starch-based composite specimen; (**b**) engineering stress–strain curves of the corn-starch-based composite specimen; (**c**) Young’s-modulus curves of the corn-starch-based composite specimen; (**d**) force–displacement curves of the orange-peel-gelatin-based composite specimen; (**e**) engineering stress–strain curves of the orange-peel-gelatin-based composite specimen; (**f**) Young’s-modulus curves of the orange-peel-gelatin-based composite specimen. Mechanical tests were conducted with duplicate specimens per material (n = 2); individual curves for each replicate are shown in panels (**a**–**f**). For the corn-starch-based composite, the peak force, tensile strength, and failure displacement were 4.70 ± 0.07 N (CV = 1.5%), 4.35 ± 0.23 MPa (CV = 5.4%), and 12.2 ± 1.5 mm (CV = 12.4%), respectively. For the orange-peel-gelatin-based composite, the corresponding values were 1.18 ± 0.03 N (CV = 2.3%), 0.79 ± 0.07 MPa (CV = 8.6%), and 35.6 ± 5.6 mm (CV = 15.8%), respectively.

Owing to the manual fabrication of the biobased composite specimens and the exploratory nature of this screening, two replicates were tested per material system. Results are reported as mean ± standard deviation with the coefficient of variation. No inferential statistical analysis was conducted due to the limited number of replicates. All mechanical trends obtained from limited replicates are preliminary and qualitative. All mechanistic interpretations in this work are inferred from macroscopic tensile data without direct microstructural verification (e.g., SEM, FTIR), and thus remain tentative. The basic mechanical responses of corn-starch-based composites and orange-peel-gelatin-based composites are shown in Figure 11. The specimens were prepared according to the baseline parameters described in Section 2.2. The force–displacement curves of the corn-starch-based composite showed a nonlinear response followed by brittle fracture. Peak forces (approximately 4.7 and 4.6 N) and failure displacements (approximately 11.1 and 13.3 mm) of the two tests were similar (Figure 11a). The corresponding stress–strain curves gave tensile strengths of approximately 4.5 MPa and 4.2 MPa, respectively. Regarding tensile behavior, the material showed brittle fracture without any significant yield before the peak stress. The failure mechanism is characterized by a sudden fracture at the maximum load. This is only for illustration, but its tensile brittle-fracture mechanism (sudden failure without significant pre-yield stretching) is similar to that of the dry, inflexible paperboard we encounter in everyday life. This analogy demonstrates only the qualitative failure characteristics, not the quantitative magnitude or specifications of a given commercial paperboard grade. The instantaneous Young’s modulus of the maize-starch-derived composite is shown in Figure 11c. The curves climbed rapidly to a preliminary maximum, then declined slowly, finally falling abruptly to zero at the point of failure. During the rising phase, the two curves followed similar trajectories, which may suggest comparable initial stiffness at low strain. The initial stiffness at small strain was nearly constant. The measured stresses and strains at the summit were comparable for both specimens. Finally, in the steep drop, the failure strain of Sample 1 (about 0.25) was slightly worse than that of Sample 2 (about 0.28). This minor difference was recorded between the two specimens in the limited test set.

The force–displacement curve of the orange-peel-gelatin composite exhibited an initial elastic region with a continuous increase in force, followed by a region of a relatively stable force with a progressive increase in deformation. This observation points to ductile deformation behavior. The two samples had similar peak forces of about 1.2 N, and their failure displacements were about 39.6 mm and 31.6 mm, respectively (Figure 11d). The respective stress–strain curves showed tensile strengths of about 0.7 MPa and 0.8 MPa, respectively. Tensile properties of the orange-peel-gelatin composite showed a negligible yield strength, followed by a long range with a relatively constant stress and a large strain range. This tensile behavior, as observed in the present limited tests, may show features reminiscent of materials used for cushioning and energy absorption; however, such observations cannot be used to validate practical application suitability. For illustrative purposes only, its tensile deformation behavior resembles that of common thick household rubber gloves. This qualitative comparison refers only to deformation characteristics and does not imply matching mechanical performance of a specific commercial product.

Figure 11f shows the instantaneous Young’s modulus of the orange-peel-gelatin-based composite. Both curves drop rapidly at first and then decline slowly with increasing strain. The initially high modulus reflected the material stiffness at small strain. The sharp drop in modulus at low strain may suggest that the material can undergo large deformation under applied load. The gradual reduction of modulus at later stages reflects a progressive loss of deformation resistance before fracture. The modulus finally falls to zero at different failure strains, while the overall failure behavior remains similar.

#### 3.2.2. Effects of Key Formulation Parameters on Mechanical Properties

In this section, the effect of important formulation parameters on mechanical properties relevant to the initial choice of materials is examined after the effective preparation of composites based on corn starch and orange-peel gelatin. A univariate experimental design was used to evaluate the effect of corn starch, glycerol, and white vinegar concentrations on the tensile strength, elongation at break, and elastic modulus for the corn-starch-derived composite. These results give a basis for a first formulation evaluation. The evaluated concentrations of corn starch, glycerol, and white vinegar are shown in Table A1.

**Figure 12:**
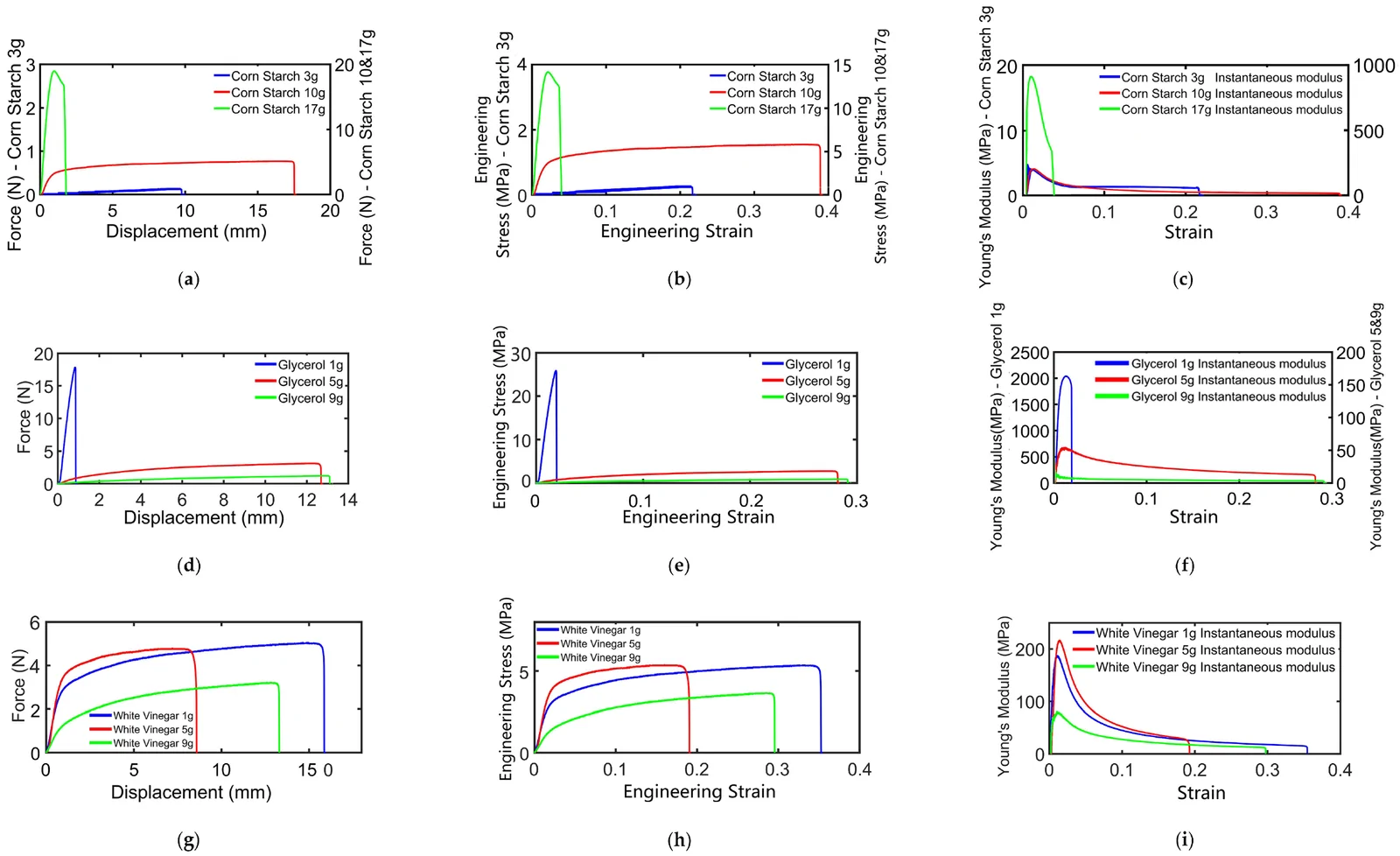
Mechanical properties of corn-starch-based composites at different formulation levels. (**a**) Force–displacement curves of corn-starch-based composite samples with different starch contents; (**b**) engineering stress–strain curves of corn-starch-based composite samples with different starch contents; (**c**) Young’s-modulus curves of corn-starch-based composite samples with different starch contents; (**d**) force–displacement curves of corn-starch-based composite material samples with different glycerol contents; (**e**) engineering stress–strain diagrams of corn-starch-based composite material samples with different glycerol contents; (**f**) Young’s-modulus diagrams of corn-starch-based composite material samples with different glycerol contents; (**g**) force–displacement curves of corn-starch-based composite material samples with different white vinegar contents; (**h**) engineering stress–strain diagrams of corn-starch-based composite material samples with different white vinegar contents; (**i**) Young’s-modulus diagram of corn-starch-based composite material samples with different white vinegar contents. Each parameter level was tested with a single specimen (n = 1) to investigate the effect of formulation variation on mechanical properties. The reference basic corn-starch-based composite formulation (tested in duplicate, n = 2) is presented in Figure 11. Quantitative values for all formulations are summarized in Table 2.

The mechanical responses obtained by the individual variation of concentrations of maize starch (CS, 3–17 g), glycerol (GL, 1–9 g), and white vinegar (WV, 1–9 g) are summarized in Figure 12. The effect of maize-starch concentration (CS, 3–17 g) is studied first. The force–displacement curve for the 3 g starch specimen shown in Figure 12a shows the minimum force level that gradually increases with displacement. This observation may reflect the lowest deformation resistance and strength among the three formulations. The force reaction of the 10 g specimen of starch is average. The force curve of the 17 g starch specimen shows a rapid increase in force to a maximum during the first stage of the displacement loading, followed by a sharp decrease, which is characteristic of brittle fracture. This mechanical response could be related to the development of a more compact network structure at high levels of starch. The stress–strain curves of composite samples prepared from maize starch with different starch concentrations are shown in Figure 12b. The 3 g starch sample has the lowest tensile stress and slow growth of strain. This behavior is hypothesized to originate from weak intermolecular forces at low starch levels that may prevent the formation of an efficient stress-transfer network and lead to lower strength. The 17 g starch sample shows a rapid increase in stress with small strain. The phenomenon suggests that high starch content may result in significantly increased hydrogen bonding and physical cross-linking density to allow the material to attain considerable strength at very low strain. But this suggested mechanism is inferred from macroscopic assessments, not from direct microscopic observations. Figure 12c shows Young’s modulus obtained from the linear part of the stress–strain curves. The large fluctuations in the immediate modulus at low strain, especially the large initial peak for the 17 g specimen, may be due to measurement artifacts such as initial specimen positioning, machine compliance, or uneven contact, rather than being an intrinsic material property. Therefore, the stiffness assessment in this work is mainly based on the overall gradient of the stress–strain curve in the approximately linear region instead of the detailed shape of the instantaneous modulus curve. Here, the 3 g starch sample is the least rigid, correlating with its lower strength and higher elongation. The 17 g sample, however, has a steeper initial gradient, consistent with its greater strength and brittle fracture. In general, increasing the starch concentration from 3 g to 17 g raised recorded force, stress, and initial stiffness. However, the material deformability and toughness gradually declined. This observed trend may suggest a transition in mechanical behavior from high toughness and low strength toward high strength and low toughness within the tested formulation range. These conclusions are drawn primarily from the force–displacement and stress–strain curves; the instantaneous modulus data are interpreted with caution due to potential artifacts at small strains.

**Table 2:** Mechanical properties of corn-starch-based composites under different formulation parameters.

Varied Parameter	Level	Peak Force (N)	Tensile Strength (MPa)	Failure Displacement (mm)	Fracture Strain (%)
Corn starch content	3 g	0.138	0.274	10.012	22.248
Corn starch content	10 g	5.120	5.818	17.641	39.201
Corn starch content	17 g	18.966	14.112	1.835	4.079
Glycerol content	1 g	17.848	25.942	0.918	2.040
Glycerol content	5 g	3.119	2.766	12.756	28.347
Glycerol content	9 g	1.217	0.887	13.241	29.424
White vinegar content	1 g	5.042	5.363	15.954	35.453
White vinegar content	5 g	4.786	5.414	8.646	19.212
White vinegar content	9 g	3.217	3.656	13.413	29.807

Note(s): Each formulation condition was tested with a single specimen (n = 1), serving for trend analysis only.

For the glycerol series (GL, 1–9 g), Figure 12d shows the force–displacement curves of corn-starch-based composite samples with different glycerol contents. For the 1 g glycerol sample, the force rapidly increases to a peak value (approximately 18 N) at low displacement (0–2 mm), which may suggest high load-bearing capacity. However, the subsequent rapid decline in the curve may also suggest a brittle fracture response. In contrast, the force of the 9 g glycerol sample increases only mildly with increasing displacement, with a much lower peak value of about 2 N, which may suggest a substantially reduced mechanical strength. This comparison of the force–displacement curves shows that samples with lower glycerol content may exhibit greater resistance to deformation, corresponding to a harder material response. Figure 12e shows the stress–strain curves of corn-starch-based composite samples with different glycerol contents. For the 1 g glycerol sample, the stress rapidly reaches a peak value (approximately 25 MPa) at very low strain (<0.05). The higher tensile strength could be tentatively attributed to the formation of a dense, rigid three-dimensional network structure between the molecular chains of the biopolymer, which provides more tensile resistance. The plastic deformation region is almost absent and reconfirms the typical brittle high-strength character of the material. Conversely, the 9 g glycerol sample exhibits very low stress levels (<2 MPa) over the entire range, with no significant increase in stress with strain. This means that the strength has been considerably weakened. The samples with less glycerol had higher stress for a certain strain, which may suggest that they are stiffer and stronger. The initial Young’s modulus was calculated from the linear region of the stress–strain curves as shown in Figure 12f. The 1 g glycerol sample shows a very high initial Young’s modulus, while the 9 g glycerol sample shows a much lower initial Young’s modulus and apparent stiffness. This observation may reflect material softening at higher glycerol loading. Increasing glycerol content appeared to reduce the tensile strength and initial stiffness of starch composites while enhancing ductility, which suggests a plasticizing effect within the tested concentration range.

Figure 12g shows the force–displacement curves of corn-starch-based composites with different white vinegar contents in the white vinegar series (WV, 1–9 g). The force is maximum for the 1 g white vinegar sample and minimum for the 9 g sample at the same displacement; the 5 g sample has an intermediate value. This tendency may suggest that increasing the white vinegar content will decrease the load-bearing capacity. Figure 12h presents the stress–strain curves of corn-starch-based composite samples with different white vinegar contents. The 1 g white vinegar sample also shows the steepest initial slope of the linear region and the greatest stress. This may reflect that it has a greater initial stiffness and bearing strength. In contrast, the lowest values of both parameters are obtained for the 9 g sample. These observations appear to indicate that increasing the white vinegar content reduces both the initial stiffness and ultimate strength of the material, making large deformation more likely at low stress. The 1 g white vinegar sample has the highest strength value, whereas the 9 g white vinegar sample shows a considerable strength decrease. Therefore, the inclusion of white vinegar may weaken the composite structure and lower its strength. The Young’s modulus was calculated from the linear part of the stress–strain graphs (see Figure 12i). The 1 g white vinegar sample shows the highest initial modulus value in the preliminary low-strain stage, which is in sharp contrast to the 9 g sample, which shows the lowest value. The stress drops rapidly after the peak in the linear phase of the 1 g white vinegar specimen, but the stress drops slowly in the case of the 9 g white vinegar specimen. Thus, increasing the concentration of white vinegar not only decreases the initial stiffness but also seems to change the strain-dependent mechanical response. Regarding strength retention, the high-vinegar formulation, although initially less rigid, may allow for a more gradual stress dissipation during deformation due to the slower modulus reduction, thus preventing the sudden drop in load-bearing capacity seen in the 1 g sample. These results provide the first guidance in tuning mechanical properties by the concentration of white vinegar.

For the orange-peel-gelatin-based composites, the effects of orange-peel powder, glycerol, and gelatin contents on mechanical properties were likewise investigated using a univariate experimental design. The tested levels of orange-peel powder, glycerol, and gelatin are provided in Table A2.

**Figure 13:**
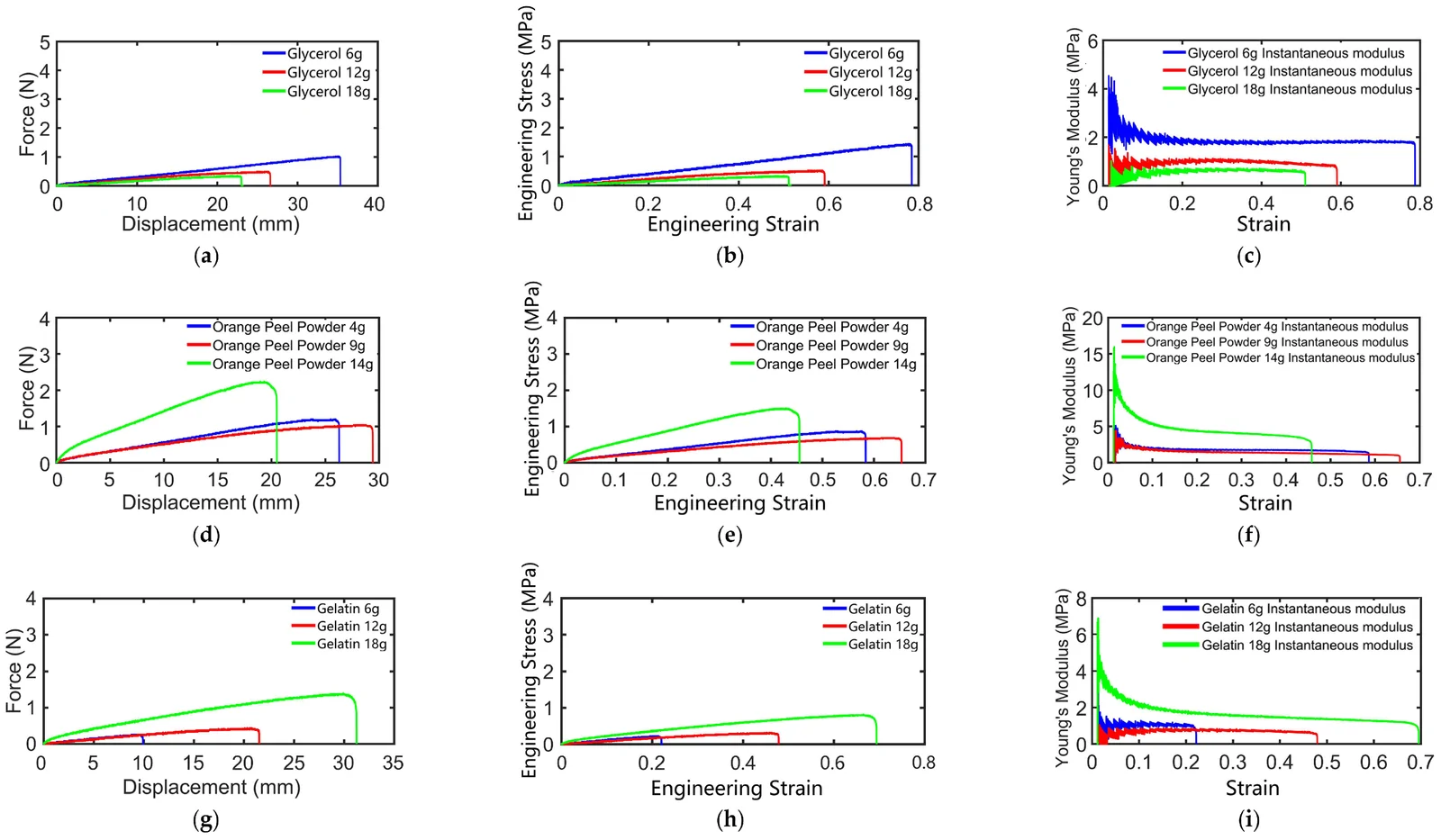
Mechanical properties of orange-peel-gelatin-based composites at different formulation levels. (**a**) Force–displacement curves of orange-peel-gelatin-based composites with different glycerol contents; (**b**) engineering stress–strain curves of orange-peel-gelatin-based composites with different glycerol contents; (**c**) Young’s-modulus curves of orange-peel-gelatin-based composites with different glycerol contents; (**d**) force–displacement curves of orange-peel-gelatin-based composite materials with different orange-peel powder contents; (**e**) engineering stress–strain diagrams of orange-peel-gelatin-based composite materials with different orange-peel powder contents; (**f**) Young’s-modulus diagrams of orange-peel-gelatin-based composite materials with different orange-peel powder contents; (**g**) force–displacement curves of orange-peel-gelatin-based composite materials with different gelatin contents; (**h**) engineering stress–strain diagrams of orange-peel-gelatin-based composite materials with different gelatin contents; (**i**) Young’s-modulus diagrams of orange-peel-gelatin-based composite materials with different gelatin contents. Each parameter level was tested with a single specimen (n = 1) to investigate the effect of formulation variation on mechanical properties. The reference basic orange-peel-gelatin-based composite formulation (tested in duplicate, n = 2) is presented in Figure 11. Quantitative values for all formulations are summarized in Table 3.

Figure 13 summarizes the mechanical responses of formulations in which glycerol (GL, 6–18 g), orange-peel powder (OPP, 4–14 g), and gelatin (GE, 6–18 g) were varied individually. For the glycerol series (GL, 6–18 g), as shown in Figure 13a, force generally increased with displacement before reaching a plateau or changing slope at larger displacements. With increasing displacement, the force of the 6 g glycerol sample reaches its highest value and exhibits the largest displacement range. The 12 g glycerol sample shows an intermediate force peak and the smallest displacement range. The 18 g glycerol sample shows the minimum force peak with a small displacement range. At equivalent displacement, specimens with higher glycerol loading recorded lower force magnitudes, which may reflect a gradual reduction in tensile resistance with increasing glycerol content under the present test conditions. Figure 13b shows the stress–strain curves of the orange-peel gelatin composites with different glycerol contents. The sample of 6 g glycerol exhibits the highest stress peak (about 2 MPa) and the largest strain range. The specimen of 12 g glycerol exhibits a moderate peak stress and strain range. The 18 g glycerol sample has the lowest peak stress at less than 1 MPa and the smallest change of strain. The recorded variations in peak stress and maximum strain may suggest that increased glycerol content correlates with reduced tensile capacity and altered deformation behavior. The observed trends could be tentatively interpreted as glycerol exerting a plasticizing effect that facilitates molecular-chain mobility while potentially weakening internal structural cohesion, thereby lowering load-bearing performance. The Young’s modulus (Figure 13c) calculated from the linear part of the stress–strain curves shows that the initial peak (around 4 MPa) is obtained by the 6 g glycerol specimen, followed by the 12 g and 18 g glycerol specimens with decreasing initial modulus values. This trend implies a possible decline in initial stiffness with increased glycerol addition. The overall trends in peak stress and initial modulus suggest that glycerol addition modifies the mechanical response of the material. The mechanical response shifts from a relatively strong and tough state with high stiffness, high strength, and large deformation capacity toward a softer state with lower stiffness, reduced strength, and restricted deformation capacity. Although the 18 g glycerol sample retains some ductile behavior, it displays a pronounced reduction in load-bearing strength.

**Table 3:** Mechanical properties of orange-peel-gelatin-based composites under different formulation parameters.

Varied Parameter	Level	Peak Force (N)	Tensile Strength (MPa)	Failure Displacement (mm)	Fracture Strain (%)
Glycerol content	6 g	1.021	1.450	35.566	79.035
Glycerol content	12 g	0.491	0.514	26.715	59.367
Glycerol content	18 g	0.354	0.330	23.238	51.641
Orange-peel powder content	4 g	1.197	0.865	26.350	58.555
Orange-peel powder content	9 g	1.040	0.686	29.475	65.500
Orange-peel powder content	14 g	2.237	1.507	20.548	45.662
Gelatin content	6 g	0.256	0.224	9.981	22.180
Gelatin content	12 g	0.432	0.319	21.887	48.638
Gelatin content	18 g	1.393	0.818	31.302	69.560

Note(s): Each formulation condition was tested with a single specimen (n = 1), serving for trend analysis only.

The force–displacement curves of gelatin-based composite samples with different concentrations of orange-peel powder (OPP, 4–14 g) are shown in Figure 13d. For all formulations, force increased to a maximum before declining with further deformation. Within the tested range, higher orange-peel powder contents generally corresponded to higher peak force values, which may indicate improved load-bearing behavior under these specific test conditions. The stress–strain behavior of the gelatin composites prepared from orange peel with different concentrations of orange-peel powder is shown in Figure 13e. The orange-peel powder of 14 g shows the highest peak stress (nearly 2 MPa) and the highest strain range. For the formulations with lower filler loading, the values decrease gradually, with the 9 g sample showing moderate values and the 4 g giving the lowest peak stress (~1 MPa). The overall trend of increased peak stress and extended deformation at higher filler loading may tentatively suggest a reinforcing effect from orange-peel powder, potentially improving the stress tolerance of the composite matrix. Young’s modulus was calculated from the linear portion of the stress–strain curves (Figure 13f). The 4 g sample of orange-peel powder has the lowest initial modulus at about 5 MPa, and the sample of 14 g has the highest, almost 15 MPa. This may suggest a possible increase.

The force–displacement graphs of composites prepared from orange-peel gelatin with various gelatin concentrations in the gelatin series (GE, 6–18 g) are shown in Figure 13g. In all graphs, the force is positively correlated with the displacement. The curves increase as the gelatin loading increases. The 18 g gelatin specimen requires the most force at the same displacement, while the 6 g gelatin specimen requires the least amount of force. This may suggest a possible increase in deformation resistance with rising gelatin content within the tested range. The 6 g gelatin specimen, shown in Figure 13h, is characterized by low stress levels and a low peak stress, resulting in a breakdown at relatively low strain. The 12 g gelatin sample shows a moderate stress and ultimate strength. The 18 g gelatin sample shows the maximum peak stress. This macroscopic tendency may support the tentative interpretation that gelatin functions as the primary film-forming matrix, and higher gelatin content could enhance the structural integrity and load-bearing capacity of the composite system. Initial Young’s-modulus values (Figure 13i) increased considerably with gelatin dosage, with the 18 g group maintaining the highest stiffness throughout deformation. All specimens exhibited gradual modulus decay under continuous loading, which may reflect progressive structural softening or local structural damage during stretching. However, the 18 g gelatin sample maintains a consistently higher modulus than the 12 g and 6 g samples throughout the entire deformation process. This trend suggests that higher gelatin content is associated with greater initial stiffness and better stiffness retention during deformation, even though the absolute differences diminish at larger strains. One possible interpretation of these macroscopic trends is that increased gelatin content may promote a more extensive polymer network, which could enhance the material’s resistance to deformation and its ability to sustain stiffness under load. However, it must be emphasized that this network-based explanation remains speculative at this stage, as it is inferred solely from macroscopic mechanical testing and lacks direct microstructural confirmation (e.g., via SEM, FTIR). Accordingly, all observed mechanical trends are qualitative and valid only within the present experimental conditions, without generalized statistical or structural certainty.

### 3.3. Soil Burial Mass Loss Test and Seed Germination Test

#### 3.3.1. Mass Loss Behavior Under Soil Burial

**Figure 14:**
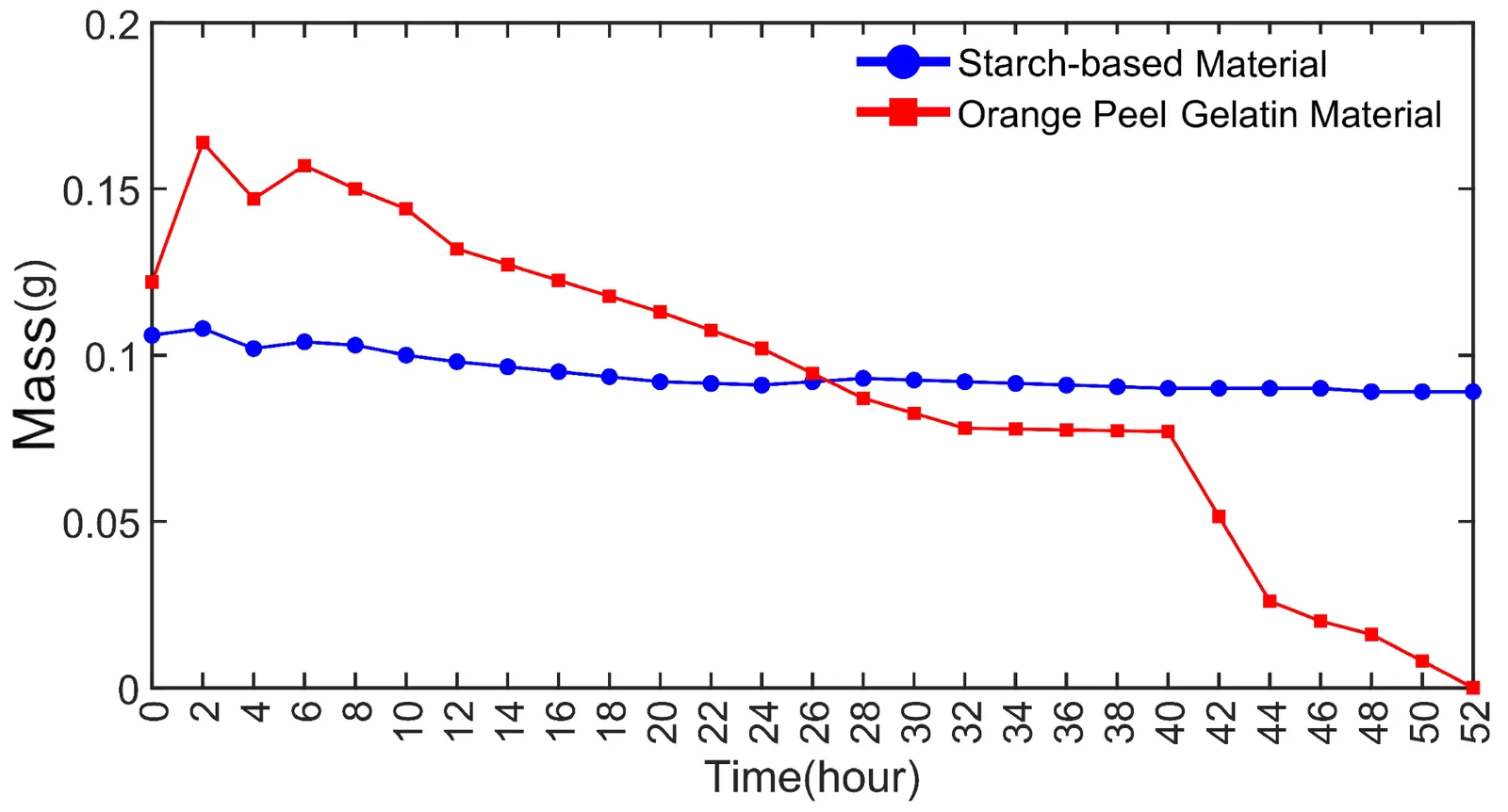
Mass variation over time for corn-starch-based and orange-peel-gelatin-based composites. Data were acquired from a single experimental trial without replicated samples.

The mass fluctuation pattern of the two composites was presented in the soil burial mass loss assessment. The results indicated that under the conditions studied, the orange-peel-gelatin composite experienced a large mass loss in 1–3 days, while the corn-starch composite experienced slow mass changes during the period of observation. The weight of the composite obtained from maize starch was constant and reduced gradually with time, as presented in Figure 14. The initial dry mass was 0.106 g, and the mass stabilized at approximately 0.09 g, with only minor fluctuations throughout the 52 h test duration. This observation may imply that the corn-starch composite maintained relatively stable mass over the test period, with limited net mass change. Such mass variation could arise from moisture exchange and gradual loss of soluble components. The mass change of the orange-peel-gelatin composite was found to be triphasic, with an initial increase and subsequent gradual and sharp decreases. In the first phase, the mass increased rapidly from 0.122 g to about 0.16 g. This might be due to the material absorption of the moisture in the early stage, leading to an increase in mass. An initial augmentation may be produced by the absorption of water. In the early stages of soil burial, water uptake may cause a temporary increase in the weight of the specimen. Subsequently, as the polymer chains hydrolyze and low-molecular-weight products are leached, the mass gradually decreases [61]. From the peak, the mass decreased gradually, likely owing to the gradual release of water and soluble fractions from the specimen. In the later stage (40–52 h), the mass rapidly decreased from approximately 0.08 g to near zero, indicating pronounced mass loss during this interval. In summary, the corn-starch-based composite maintained stable mass with only slight changes over 52 h, which may be beneficial for scenarios requiring mass retention. In contrast, gelatin composites based on orange peel are characterized by fast mass loss under soil burial conditions. It is crucial to understand that the reduction in mass may be due to the combined effects of water-soluble leaching, hydrolysis, and microbial activity, and not only to microbial biodegradation. These results reveal differences in physical stability and soil burial mass loss behavior between the two composites, which can offer a reference for material selection for potential application scenarios.

**Figure 15:**
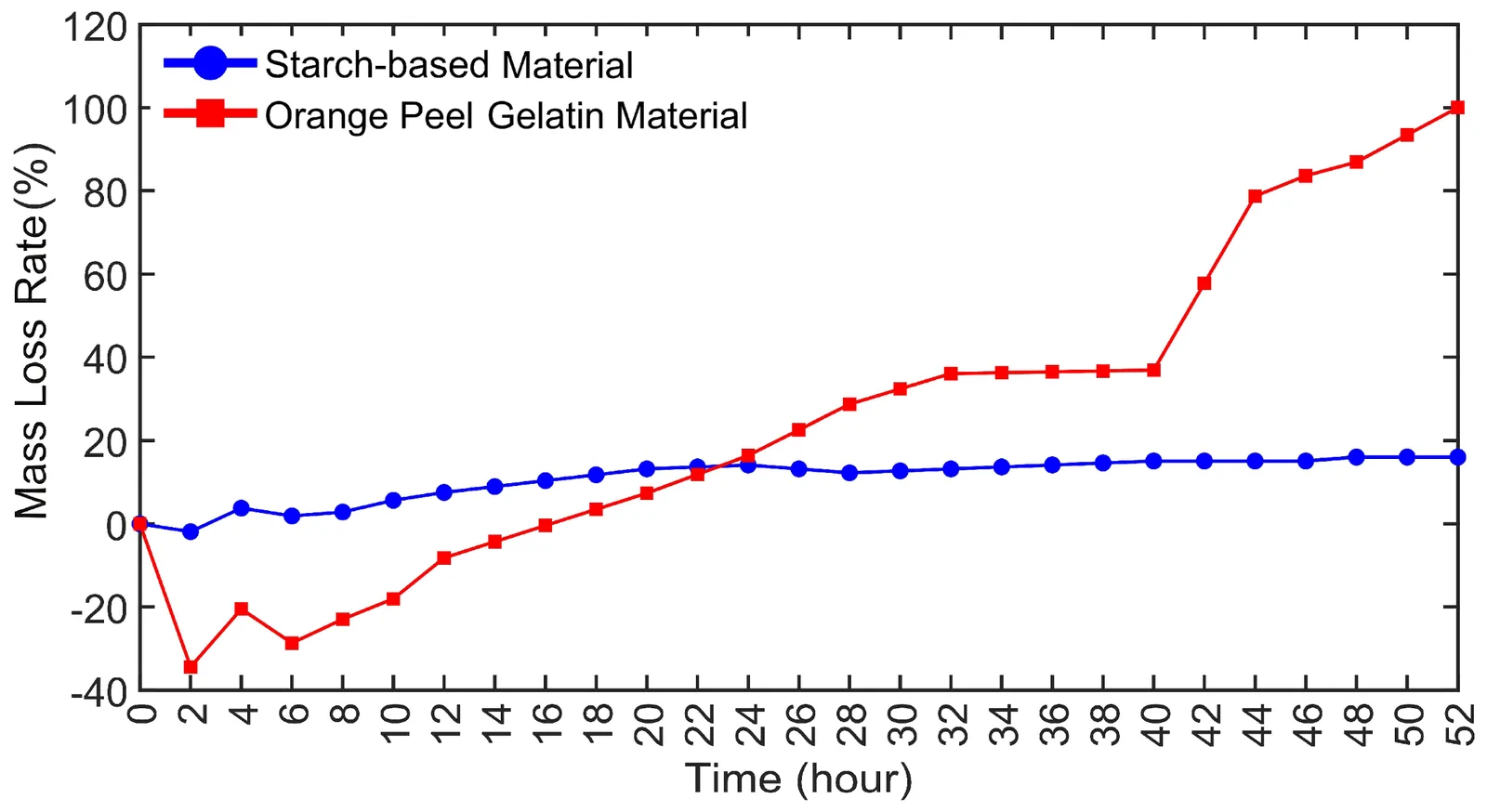
Trends in calculated mass loss rate over time for corn-starch-based and orange-peel-gelatin-based composite materials. Data were acquired from a single experimental trial without replicated samples.

As shown in Figure 15, the calculated mass loss rate of the corn-starch-based composite fluctuated slightly during the early stage and remained close to 0% overall. Temporary negative values were also observed, which can be explained by early-stage water uptake causing deviation relative to the initial dry mass. This observation may reflect that little net mass loss occurred during this period. In the middle to late stage (10–52 h), the calculated mass loss rate increased gradually, reaching approximately 18% after 52 h. The overall gradual trend may suggest a low rate of mass change and relatively high physical stability for the corn-starch-based composite under these test conditions. Similarly, the calculated mass loss rate of the orange-peel-gelatin-based composite also showed negative values in the initial stage, dropping to a minimum of approximately −35%. However, in the middle stage (10–40 h), the calculated mass loss rate shifted from negative to positive values and then increased progressively, reaching about 40% at around 40 h. During this interval, the rate of calculated mass loss continued to increase. In the later stage (40–52 h), the calculated mass loss rate increased sharply, rising from approximately 40% to around 100%, reflecting drastic mass loss behavior. The orange-peel-gelatin-based composite exhibited much more dynamic mass evolution during soil burial and a considerably higher final mass loss rate than the corn-starch-based composite, which featured slow and steady mass variation. In view of these trends, the orange-peel-gelatin-based composite may be more suitable for conceptual scenarios requiring rapid mass reduction, such as conceptual disposable packaging or tableware. For conceptual products that do not require rapid mass loss but demand a longer service life (e.g., conceptual agricultural films and slow-release packaging), corn-starch-based composite materials remain a feasible option.

#### 3.3.2. Seed Germination Performance

**Figure 16:**
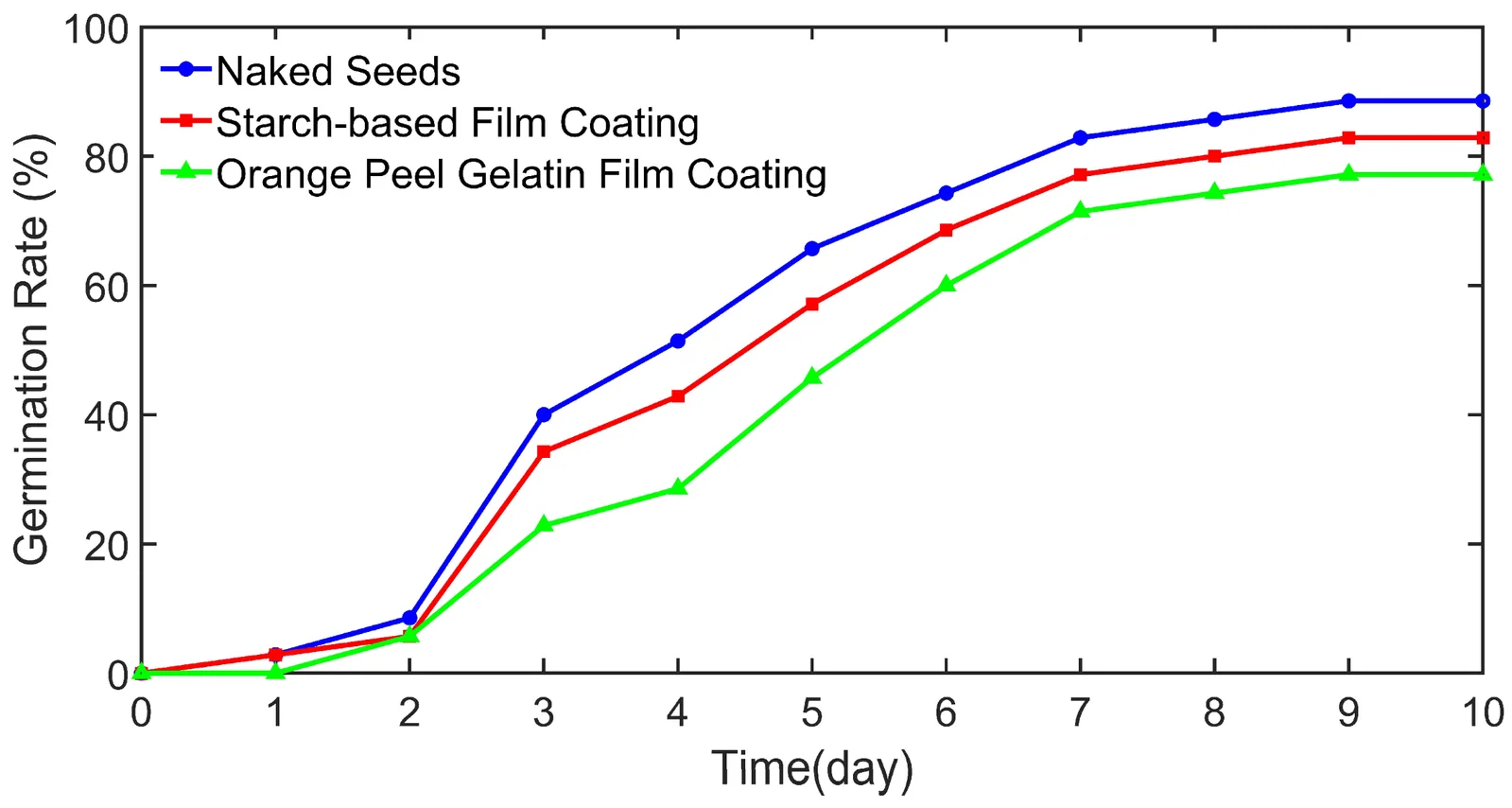
Germination-rate profiles for naked seeds and seeds coated with corn-starch-based or orange-peel-gelatin-based composite films. n = 35 seeds for each group; no independent biological replicates were performed.

The results of the germination are shown in Figure 16. During the first phase, germination was nearly absent in all three groups. This phase corresponds to the initial phases of enzyme activation, swelling, and water uptake. In the coated groups, no visible development was observed at this early stage. All three curves showed a significant increase from day 3 to day 7. The naked seeds without wrapping had the highest germination rate, followed by the seeds wrapped with the corn-starch-based composite film, and the seeds wrapped with the orange-peel-gelatin-based composite film had the lowest germination rate. Germination in all groups plateaued between days 8 and 10. The final germination percentage was about 92% for naked seeds, 86% for seeds covered with a composite material based on corn starch, and 81% for seeds covered with a composite material based on orange-peel gelatin. The highest final germination rate was observed for the uncoated control. The delayed germination was associated with both film coatings compared to the control. One tentative explanation could be that the physical barrier of the films slowed down the initial transport rates of water and oxygen and thus delayed the metabolic processes prior to germination.

Although the final germination rates of the coated groups were lower than those of the control group, they were still above 80%. Literature references for phytotoxicity screening commonly adopt a germination rate below 50% as an indicator of strong phytotoxicity, while rates exceeding 80% are regarded as indicative of low phytotoxicity [62]. However, this benchmark cannot be applied to the present dataset, as the current test was a single exploratory trial lacking independent biological replicates. The observed 6% and 11% reductions in germination rate relative to the control only reflect the numerical difference recorded under these specific test conditions. Severe toxic stress may lead to radicle necrosis, morphological abnormalities, or a sharp drop in germination rate. Within this trial, germination in both coated groups increased gradually and ultimately stayed above 80%. Seedlings exhibited normal leaf color throughout the observation period, and no obvious necrotic symptoms were noted.

The material composition provides additional context for interpreting these preliminary findings: natural polysaccharide starch is a polymer widely known to be susceptible to biological decomposition under suitable environmental conditions. Its long history of agricultural use also provides contextual support for its favorable safety profile. Gelatin in the orange-peel-gelatin-based composite film is derived from hydrolyzed collagen. The orange-peel component contains pectin, cellulose, and trace amounts of natural essential oils. Both components are derived from food byproducts. Nevertheless, it should be emphasized that this seed germination assay is only an exploratory, unreplicated trial for qualitative observation of seed germination behavior. This experiment was conducted without independent biological replicates. Therefore, the findings cannot be generalized to broader environmental conditions. In summary, under the tested conditions, the principal observed effect of corn-starch-based and orange-peel-gelatin-based composite films was delayed seed germination. Based solely on seed germination responses measured in this preliminary test, only mild inhibitory effects on seed germination were detected under the specific test conditions.

### 3.4. Conceptual Toy Design

#### 3.4.1. Design Sketch

Building-block toys generally comprise multiple small components that children handle frequently and directly. Because young children are still undergoing physiological development, their tolerance to potential hazards may be limited, and their ability to recognize and avoid external risks is not yet fully developed. These factors may increase risks such as accidental ingestion of small parts and impact-related injury during play. Accordingly, corn-starch-based and orange-peel-gelatin-based composites were selected as candidate substrate materials for the conceptual designs. The starch-based composite shows a relatively stiff response, whereas the gelatin-based composite is softer, more flexible, and more stretchable. These two materials have different properties that are relevant for the design of conceptual toys. Two opposite characteristics have been identified, for which two directions of conceptual design have been developed. The first realization of multidirectional assembly was by the development of modular building-block units. The relatively rigid starch-based composite provides structural support to the modular units, and the multidirectional geometry widens the scope of the assembly. In the second step, we combined the flexibility of the orange-peel-gelatin-based composite with origami-inspired principles to create magnetic units that can deform and stretch. This enabled a wider range of dynamic configurations and forms.

**Figure 17:**
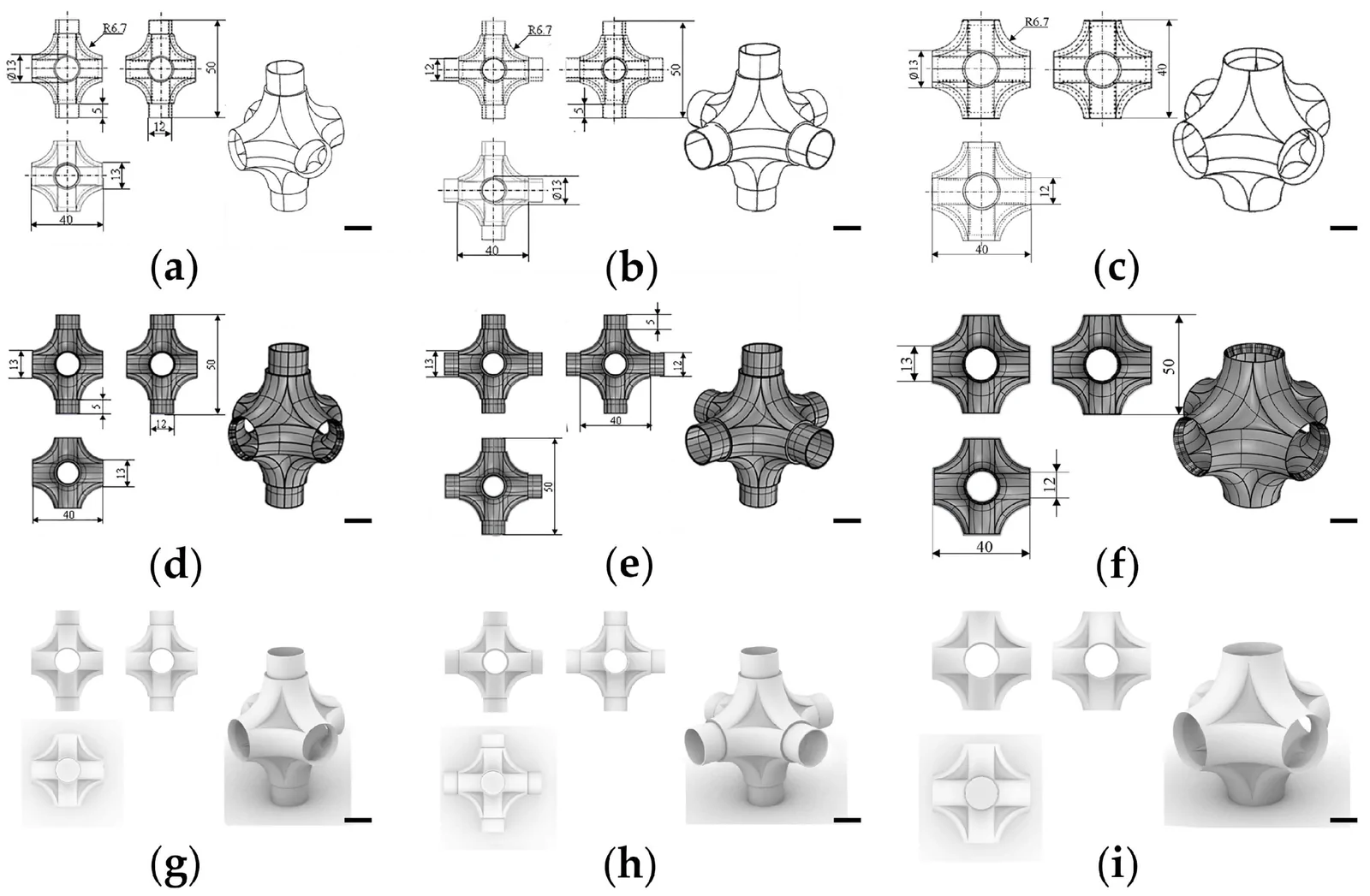
Three-view drawings of corn-starch-based composite building-block components. (**a**) Line drawing of a bidirectional extension element; (**b**) line drawing of an omnidirectional extension element; (**c**) line drawing of an omnidirectional non-extension element; (**d**) shaded drawing of a bidirectional extension element; (**e**) shaded drawing of an omnidirectional extension element; (**f**) shaded drawing of an omnidirectional non-extension element; (**g**) rendered drawing of a bidirectional extension element; (**h**) rendered drawing of an omnidirectional extension element; (**i**) rendered drawing of an omnidirectional non-extension element. Scale bar 12 mm.

After the component geometry and structure were defined, the assembly process and connection logic were further developed. The two systems were designed to support progression from basic connection operations to more complex spatial construction, as described below:

As shown in Figure 17, the assembly sequence for the corn-starch-based modular blocks follows a graded-difficulty concept intended to provide a stepwise construction pathway. In Phase I, the basic building blocks are omnidirectional non-extension components. As none of the six faces has an extension structure, these components can be combined only by simple stacking or juxtaposition. This constraint introduces fundamental concepts such as balance, stability, and spatial position. Phase II introduces directional connections using bidirectional extension elements with extension structures on both faces. Such directional restrictions allow for linear or planar construction. Assembly requires the orientation of the extension structures to be considered in advance to achieve effective connections. This stage provides a transition toward more complex spatial arrangements. In Phase III, omnidirectional extension components enable connections on all six sides and increase assembly complexity. Connections across multiple directions must then be coordinated within three-dimensional space. Assembly therefore extends beyond linear growth to consideration of spatial interference and force transmission among multiple components. This configuration permits three-dimensional structures such as frames, cage-like forms, and more complex topologies. Overall, combining the three component types provides a progressive assembly system spanning one-dimensional, planar, and three-dimensional configurations.

**Figure 18:**
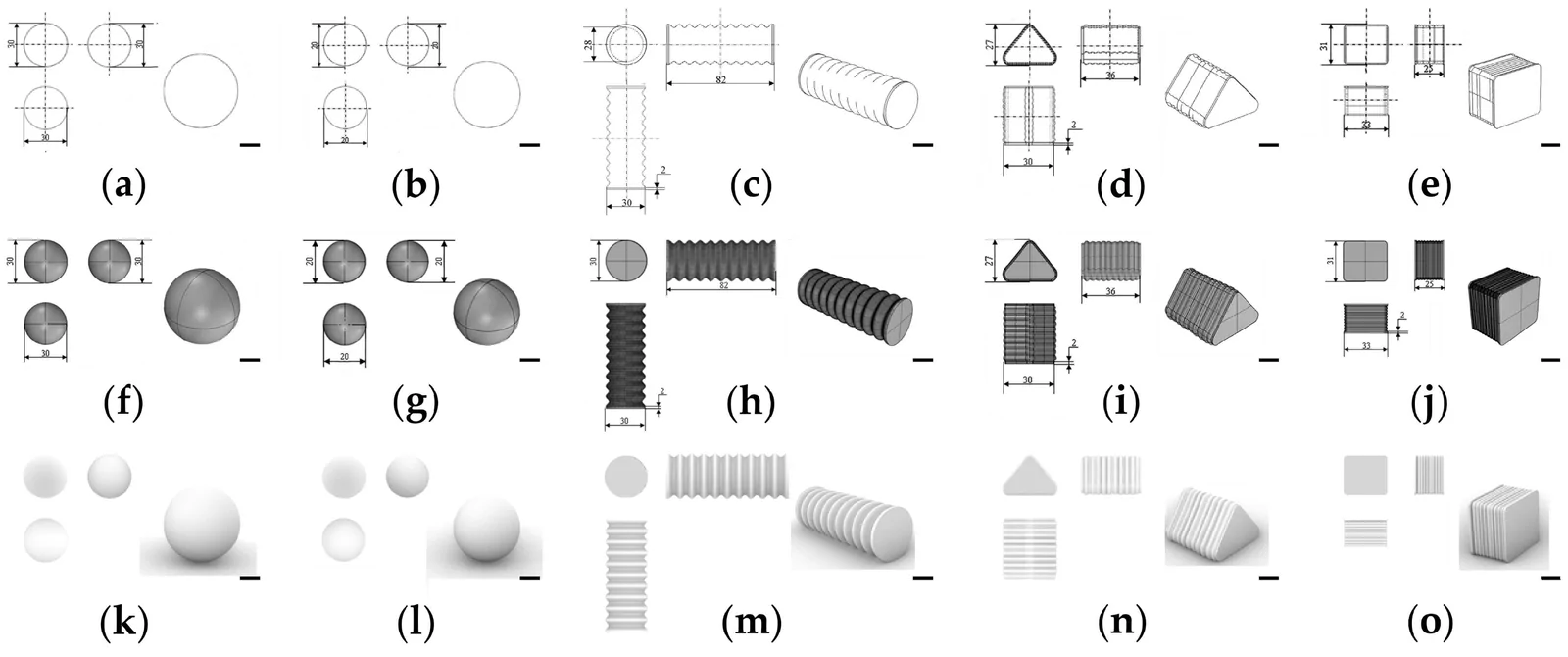
Three-view drawings of orange-peel-gelatin-based composite building-block elements. (**a**) Line drawing of a large spherical element; (**b**) line drawing of a small spherical element; (**c**) line drawing of a circular cross-section element; (**d**) line drawing of a triangular cross-section element; (**e**) line drawing of a square cross-section element; (**f**) shaded three-view drawing of a large spherical element; (**g**) shaded three-view drawing of a small spherical element; (**h**) shaded three-view drawing of a circular cross-section element; (**i**) shaded three-view drawing of a triangular cross-section element; (**j**) shaded three-view drawing of a square cross-section element; (**k**) rendered three-view drawing of a large spherical element; (**l**) rendered three-view drawing of a small spherical element; (**m**) rendered three-view drawing of a circular cross-section element; (**n**) rendered three-view drawing of a triangular cross-section element; (**o**) rendered three-view drawing of a square cross-section element. Scale bar 12 mm.

For the orange-peel-gelatin-based blocks shown in Figure 18, the assembly concept places greater emphasis on flexible and exploratory configurations. Assembly relies on magnetic attraction between components and on spherical magnets of different sizes that function as universal connectors. Different magnet sizes or quantities can be selected according to the required stability of the target form or the desired degree of joint mobility. A second design feature is the use of elements with different cross-sectional geometries (circular, triangular, square, and spherical) to enable shape adaptation. For example, a spherical magnet can connect the edges of circular and triangular elements, allowing unusual connections between different cross-sections and the creation of transitional or irregular shapes. In general, the orange-peel-gelatin-based system increases the diversity of structures and flexibility of assembly by combining rigid magnetic connectors and flexible composite components.

#### 3.4.2. Design Rendering

**Figure 19:**
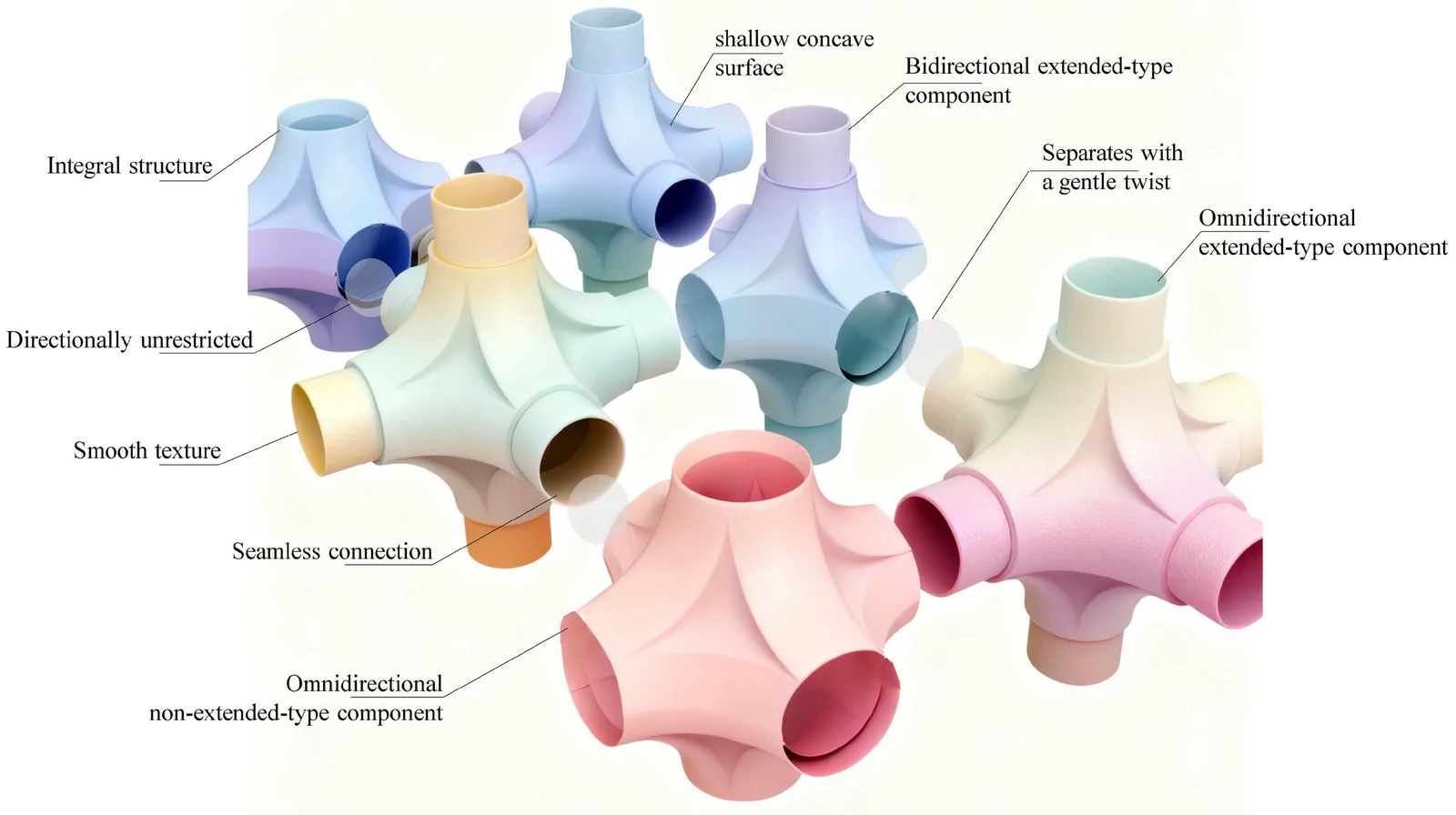
Exploded view of the corn-starch-based composite building blocks.

**Figure 20:**
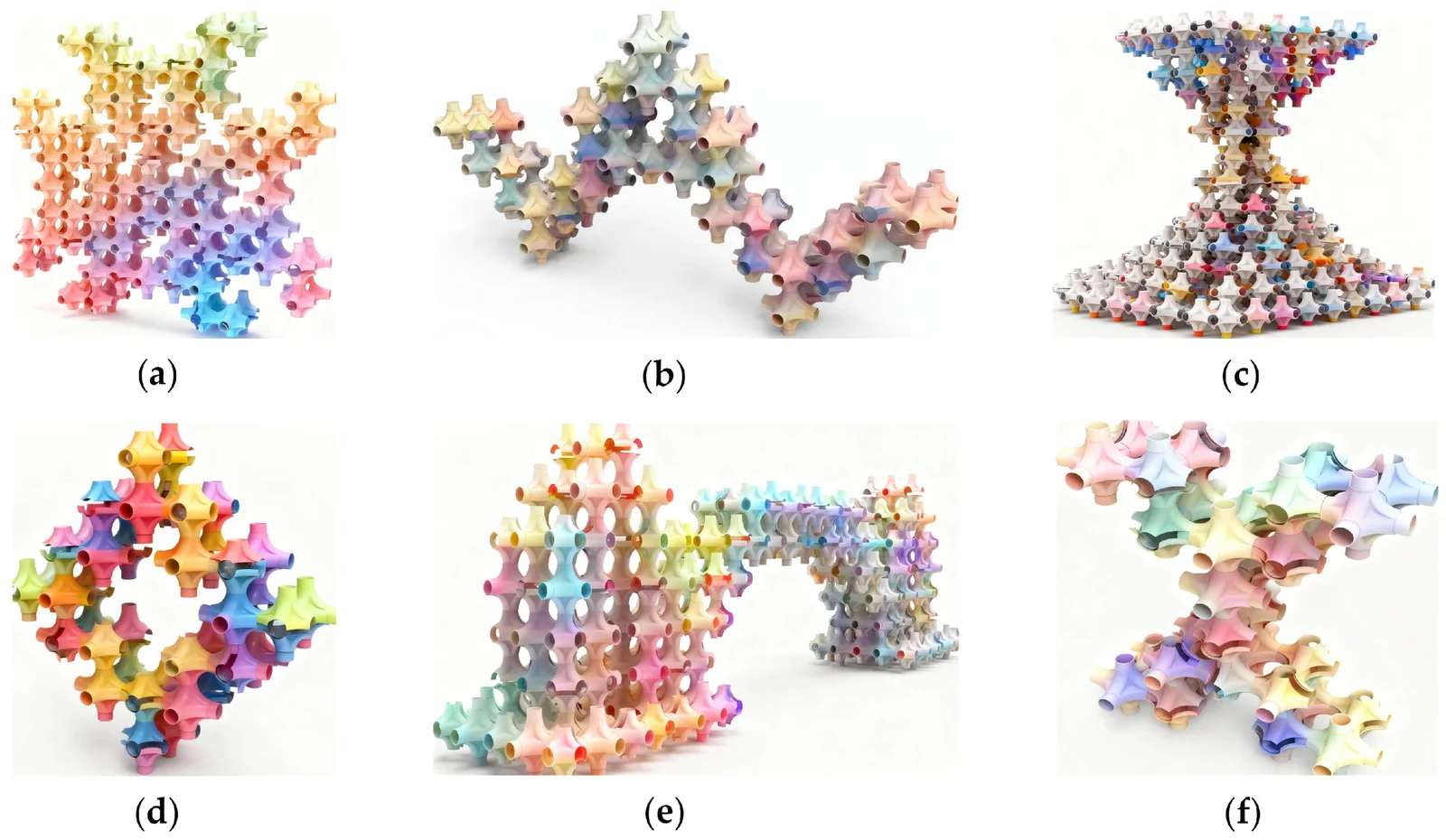
Rendered configurations of corn-starch-based composite building blocks. (**a**) Snowflake shape; (**b**) W shape; (**c**) hourglass shape; (**d**) rhombus shape; (**e**) arch shape; (**f**) cross shape.

**Figure 21:**
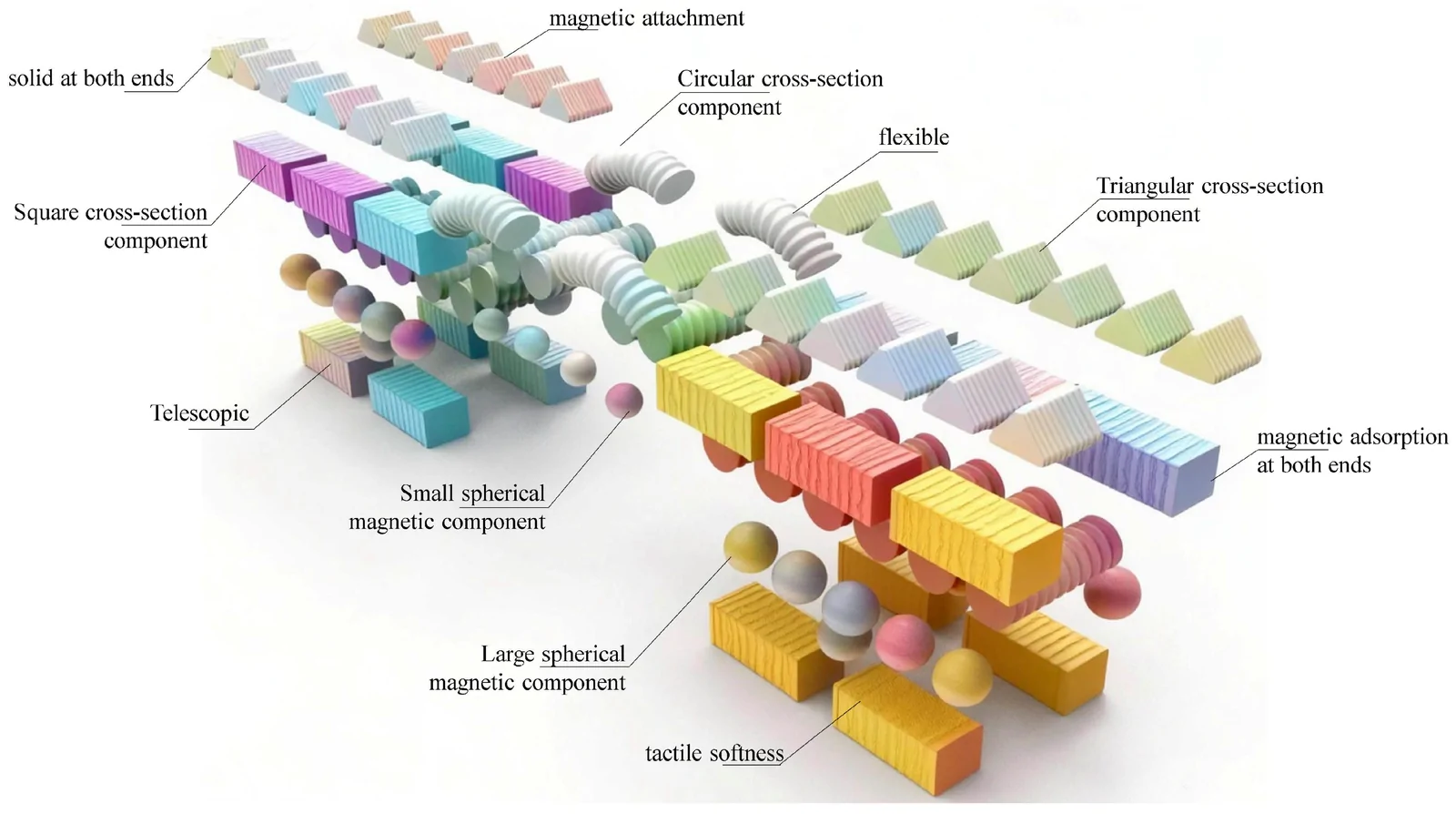
Exploded view of the orange-peel-gelatin-based composite building blocks.

**Figure 22:**
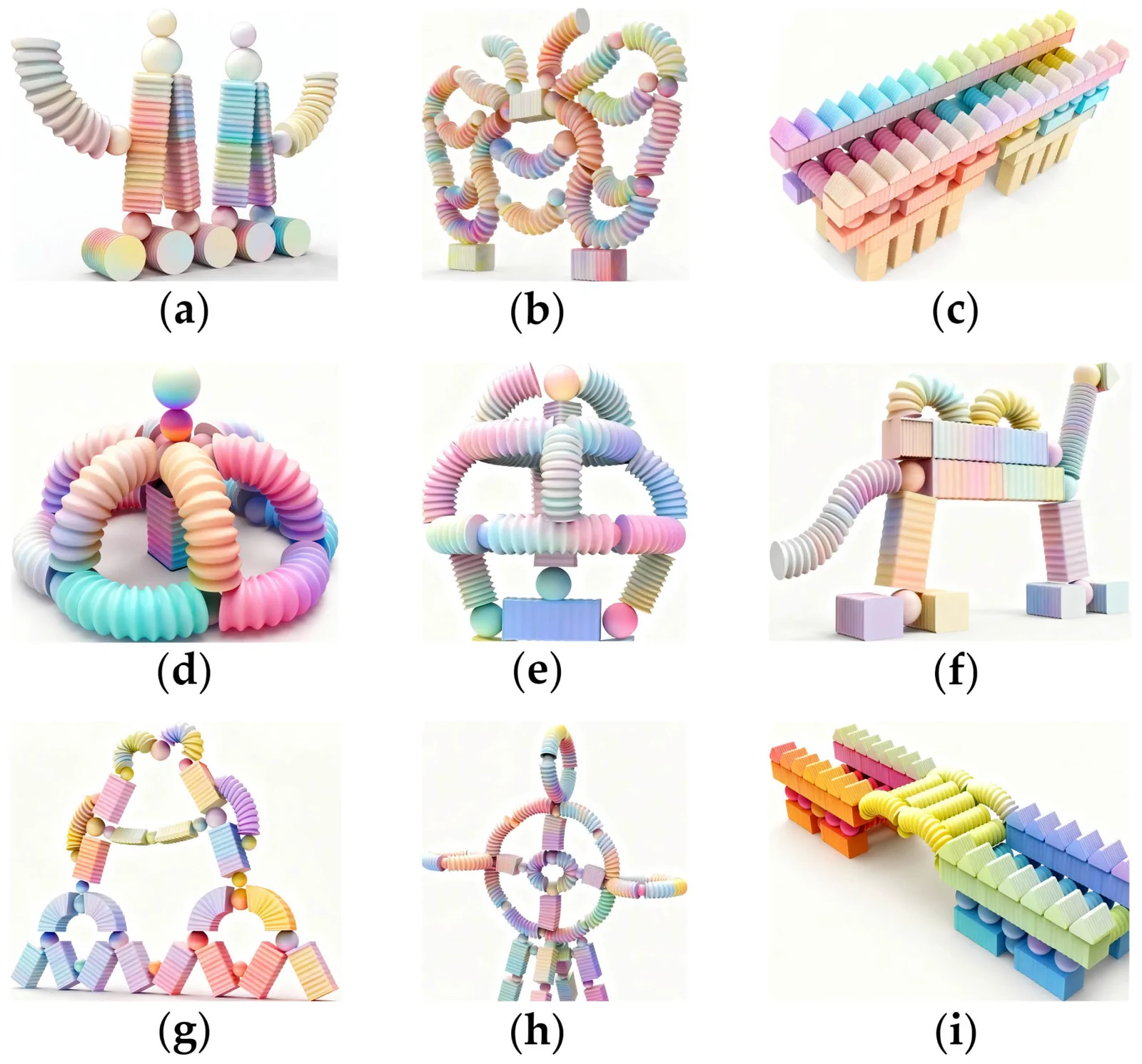
Rendered configurations of orange-peel-gelatin-based composite building blocks. (**a**) Double human shape; (**b**) butterfly shape; (**c**) railway shape; (**d**) hat shape; (**e**) ring shape; (**f**) camel shape; (**g**) frog shape; (**h**) Ferris wheel shape; (**i**) bridge shape.

**Figure 23:**
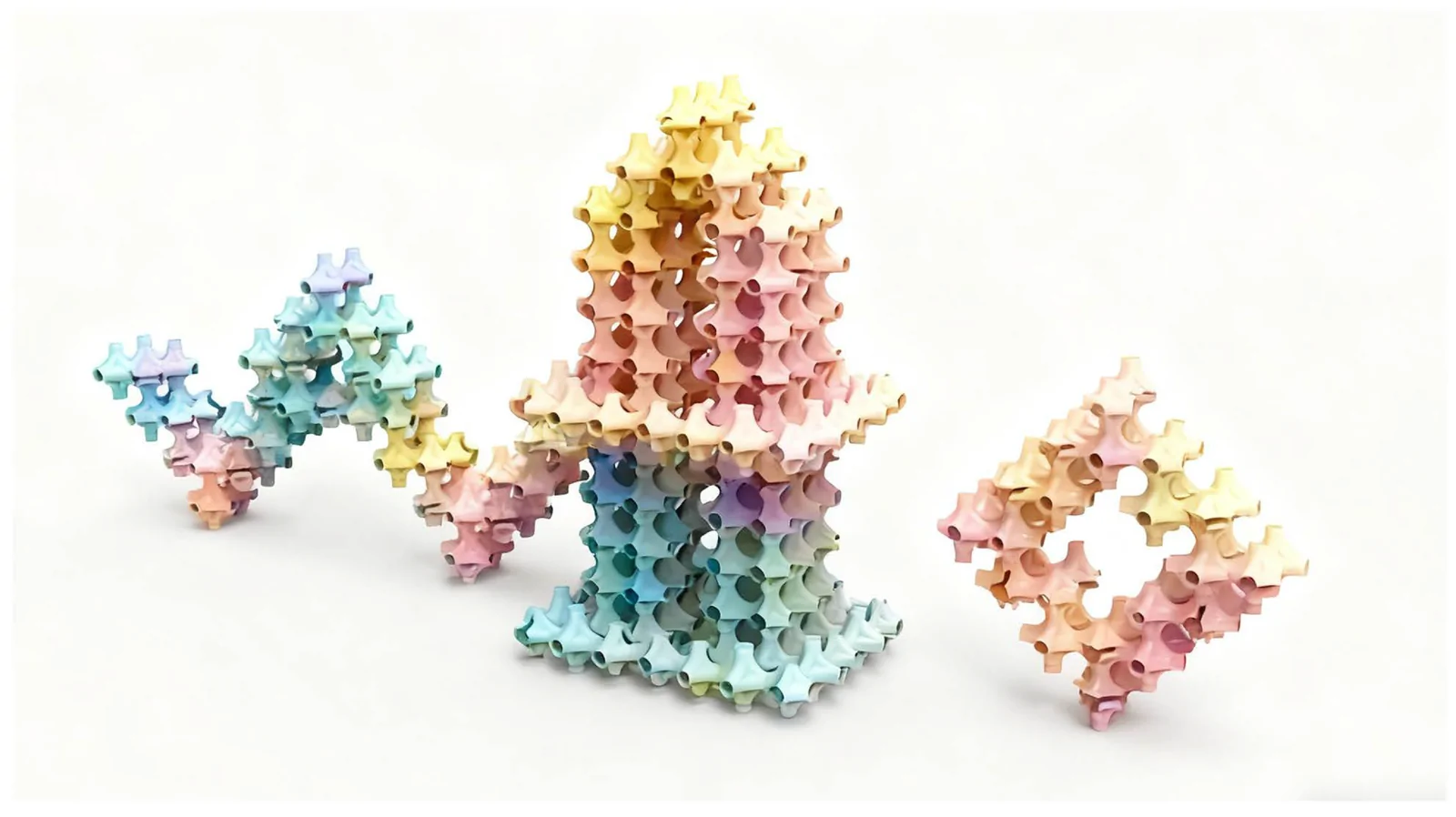
Overall rendering of corn-starch-based composite building-block toy.

**Figure 24:**
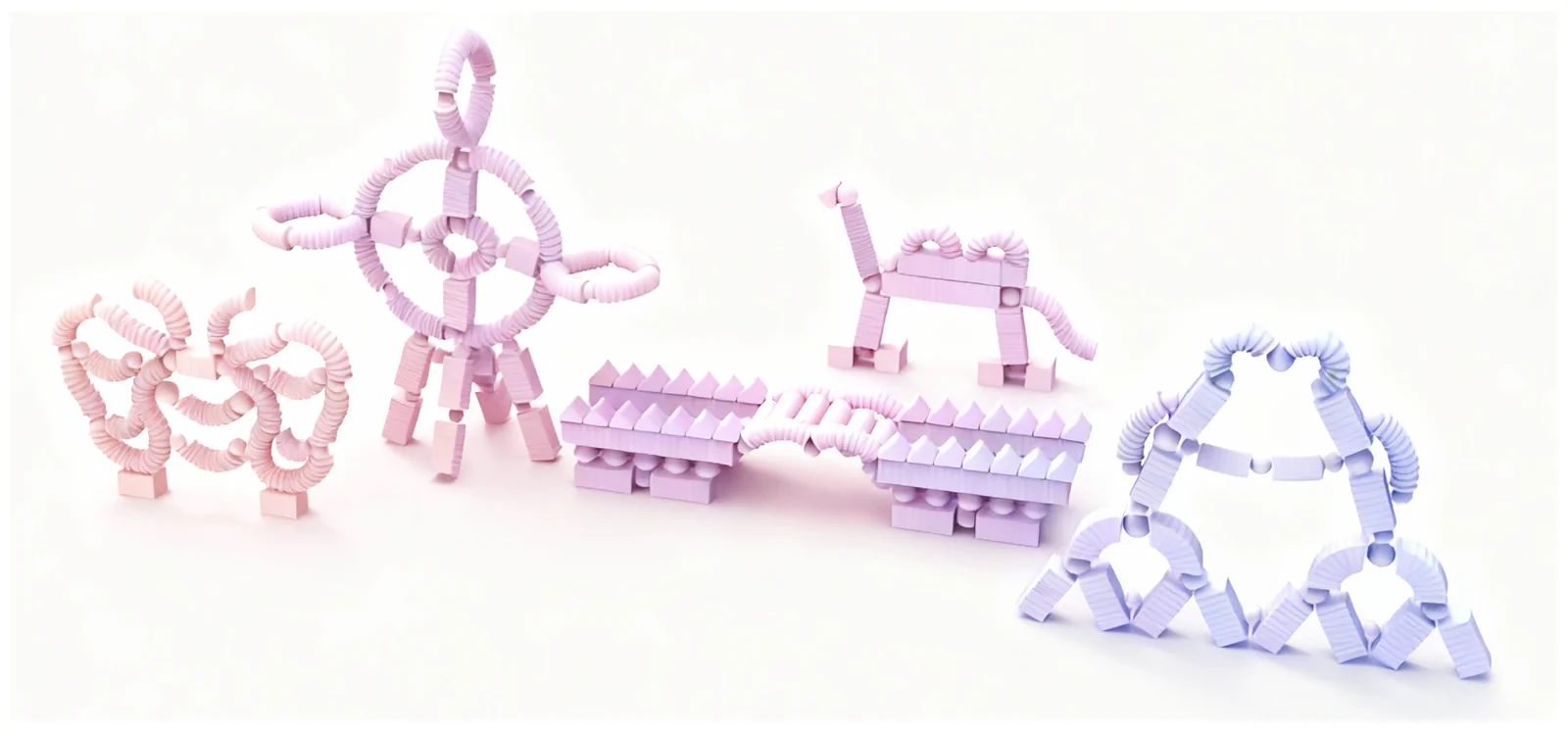
Overall rendering of the orange-peel-gelatin-based composite building-block toy.

As shown in Figure 19, the corn-starch-based building-block components are designed as integrally molded, seamless units. The configuration is used to maintain structural continuity in the assembly process. The renderings capture the desired look and feel, with proper geometry and smooth surfaces. A twist-on connection mechanism has been incorporated to make assembly and disassembly easier. Figure 20 shows various structures that can be constructed using composite building blocks from maize starch. The assemblies are based on a modular design concept based on basic units and multidimensional configurations, which shows the system’s potential for modular combination. Figure 21 presents an exploded view of the orange-peel-gelatin-based composite building blocks. The modular system is designed to be flexible by providing magnetic connections, multiple interface geometries, and functional configurations. The design can be assembled in different structural configurations by combining the magnetic connection with the material flexibility and stretchability. Figure 22 depicts the building blocks based on orange-peel gelatin in different assembling configurations. The design freedom offered by multidirectional assembly is exemplified by the ability to combine units with different cross-sectional geometries to generate increasingly complex structures. The final renderings of the composite building blocks developed are shown in Figure 23 and Figure 24. The blocks come in a colorful modular format designed to encourage sensory interaction, spatial cognition, and collaborative play for children.

## 4. Discussion

This study preliminarily explored the preparation and basic performance of starch-based and orange-peel-gelatin-based biocomposite systems, with qualitative and semi-quantitative screening of dyeing behavior, mechanical responses, soil burial mass loss characteristics, and seed germination performance.

### 4.1. Discussion of Dyeing Performance

The dyeing experiments qualitatively showed that natural plant pigments could impart color to starch- and gelatin-based composite materials, enabling the acquisition of multicolored composites covering red, orange, yellow, green, and purple hues. Visual observation confirmed that both 1 mm- and 5 mm-thick composite specimens could be successfully stained with natural plant pigments. Visual differences in color depth were observed among specimens with different thicknesses, with thicker samples appearing slightly darker upon visual inspection. On a qualitative basis, both starch- and gelatin-based composite matrices were successfully stained with the natural plant pigments, demonstrating the feasibility of imparting color to the two biocomposite systems via plant dyeing.

Previous studies have confirmed that starch- and gelatin-based composite materials contain multiple polar functional groups, including hydroxyl, carboxyl, and amino groups, that can interact strongly with various synthetic dye molecules through hydrogen bonding and electrostatic attraction [63,64,65]. Specifically, starch-based polymer materials have shown affinity for cationic dyes such as methylene blue [63], gelatin-based hydrogels have been reported to remove methylene blue effectively [64], and structural characterization of starch/gelatin composite films has confirmed the presence of the aforementioned functional groups [65]. Drawing on these published interaction mechanisms, it is tentatively hypothesized that the composite matrices may also bind natural plant pigments bearing hydroxyl and carbonyl groups (e.g., anthocyanins, carotenoids, and chlorophyll). This provides a plausible mechanistic explanation for the successful staining observed in this work, although direct experimental evidence verifying pigment–matrix binding is not available in the present study.

### 4.2. Discussion of Mechanical Properties

This study adopted a unified fabrication protocol and consistent mechanical testing conditions for starch-based and orange-peel-gelatin-based composites, enabling a controlled side-by-side comparison of their tensile behaviors under identical laboratory environments. Such direct comparative data for these two bio-based composite systems remain limited in the existing literature, where inconsistent formulation and testing conditions often hinder cross-study comparison. It should be emphasized that all mechanical trends reported in this work are derived from limited-replicate exploratory tests and serve only as preliminary qualitative references, without statistical verification. Glycerol is a good plasticizer in the starch-based system, as it can insert itself between starch molecular chains and reduce intermolecular hydrogen bonding and the energy barrier for segmental motion. This is a tentative interpretation consistent with established plasticization theory and is not directly proven by the tensile data alone. With increasing glycerol loading from 1 g to 9 g, the material experiences a smooth transition from high-strength brittle behavior to low-strength ductile performance. The measured peak stress decreases from about 25 MPa to below 2 MPa, and the initial modulus also decreases significantly. This is accompanied by a corresponding increase in elongation at break. This behavior is in agreement with previous studies on gelatin-starch blends [66,67]. In the present work, a pure starch matrix without any synthetic cross-linkers or polyelectrolytes is used. The property evolution as a function of glycerol content is almost monotonic for the composition range considered. Mechanical performance also depends on starch content. The tensile strength ranges from 0.27 MPa to 14.11 MPa with the increase of starch loading from 3 g to 17 g, consistent with the standard behavior of starch as a rigid skeletal phase for reinforcement [68]. The fracture strain is not monotonic. It first increases from 3 g to 10 g, and then drops sharply at 17 g, when the material becomes too rigid and undergoes brittle fracture with only 4.08% strain.

White vinegar shows a non-monotonic, concentration-dependent effect on the tensile response of starch-based composites. At 1 g and 5 g, tensile strength remains comparable (5.36 and 5.41 MPa), but fracture strain drops sharply from 35.45% to 19.21%, indicating that 5 g is the most brittle condition. At 9 g, strength decreases to 3.66 MPa while fracture strain recovers to 29.81%. Combined with the instantaneous modulus curves, the 1 g sample retains the highest post-yield modulus (~50–80 MPa), whereas the 5 g and 9 g samples decay to a lower residual plateau (~20 MPa). These trends cannot be assigned a simple “low-dose brittle/high-dose ductile” interpretation; the non-monotonic response suggests that acetic acid from white vinegar may interact with starch interchain hydrogen bonds in a dose-dependent manner [69,70], but the present tensile dataset (n = 1) cannot distinguish between competing molecular interpretations (e.g., preferential disruption at localized sites versus uniform network modification) [71,72,73,74]. Direct microstructural evidence from FTIR, XRD, DSC, or equivalent characterization is needed before any mechanism can be proposed. The observed non-monotonic response does, however, indicate that white vinegar dosage is a sensitive formulation parameter that warrants further investigation with replicated specimens.

For orange-peel-gelatin-based composites, gelatin constitutes the continuous matrix phase. Increasing gelatin loading from 6 g to 18 g improves tensile strength from 0.22 MPa to 0.82 MPa and simultaneously raises fracture strain from 22.18% to 69.56%. This trend is consistent with published findings [75]. This concurrent increase in strength and extensibility was observed across the full 6–18 g range.

Incorporated orange-peel powder exerts a composition-dependent effect that is not a simple monotonic reinforcement. At 4 g and 9 g, tensile strength is comparable (0.87 and 0.69 MPa), while fracture strain increases from 58.56% to 65.50%. At 14 g, tensile strength rises to 1.51 MPa (+74% relative to 4 g), but fracture strain decreases to 45.66%. Thus, the highest powder loading strengthens the material but also embrittles it; the 9 g condition offers the most ductile response. This trend is broadly consistent with reported bio-based composite film behavior [76], although the non-monotonic strength–ductility tradeoff should be noted. Whether this arises from particle–matrix interfacial stress transfer, particle agglomeration at high loading, or a combination of both cannot be resolved from tensile data alone and requires SEM, XPS, and contact-angle measurements. These results support the prospect of orange peel as an agricultural-waste filler, pending replicated testing and interfacial characterization.

Within the gelatin-based system, increasing glycerol loading from 6 g to 18 g reduces tensile strength from 1.45 MPa to 0.33 MPa and fracture strain from 79.04% to 51.64%. Compared with the starch-based system, however, the relative influence of glycerol on gelatin is weaker: gelatin strength falls by ~77% over its composition range (6–18 g), whereas starch strength falls by ~97% over its own range (1–9 g). It should be noted that the two composition ranges are not directly aligned, so the difference in relative reduction should be regarded as indicative rather than as a direct quantitative comparison. This weaker sensitivity to glycerol aligns with established polymer chemistry: starch networks are dominated by interchain hydrogen bonds susceptible to disruption by glycerol, while gelatin networks are additionally stabilized by hydrophobic interactions and chain entanglements. This interpretation is drawn from general polymer chemistry rather than direct measurements acquired in the present study and would require FTIR, DMA, or rheological testing for confirmation. These observations provide preliminary guidance for formulation screening rather than a definitive design rule; whether these tendencies persist across replicate batches remains to be verified.

### 4.3. Discussion of Soil Burial Mass Loss Test and Seed Germination Test

The soil burial test showed clear differences in mass variation under soil incubation between the two composite systems. Orange-peel-gelatin-based composites exhibited rapid mass loss throughout soil placement, consistent with the well-documented susceptibility of gelatin matrices to hydrolytic and microbially mediated decomposition reported in previous studies [77,78,79,80,81,82]. In contrast, corn-starch-based composites displayed a much slower mass reduction trend, which aligns with the formulation-dependent soil response of starch bio-based composites described in the literature [83,84,85]. Literature discrepancies in starch mass loss rates suggest that plasticizer type and compositional variables may affect material behavior under soil conditions. The microstructural origins of the observed differences in the present composites remain uncharacterized.

Seed germination tests were performed as an exploratory assay to qualitatively document early seedling growth in the presence of the composite materials. Both composite groups induced only minor growth retardation under tested conditions, without evident acute inhibitory responses, which is consistent with previous bio-based film observations [86]. Germination assays, however, only reflect short-term plant responses and cannot represent comprehensive ecological or toxicological performance.

### 4.4. Limitations and Future Work

Despite the promising fundamental properties demonstrated by the as-prepared starch- and gelatin-based composites, the present study remains an exploratory laboratory-scale investigation. Potential toy applications are therefore proposed on the basis of basic material characterization, including tensile behavior, dyeing performance, soil burial mass loss tests, and seed germination test. Consequently, several critical metrics relevant to practical toy applications and in-depth analysis of composite mechanisms have not yet been quantitatively addressed within the current research scope.

From a fundamental materials perspective, this work has examined the macroscopic tensile property variations under different filler loading ratios. However, in-depth investigation of microstructural morphology, matrix-filler interfacial interactions, and the microscale mechanisms underlying property changes caused by adjustments in filler ratio was not pursued in this study. Long-term service durability, temperature cycling resistance, and humidity-dependent mechanical stability have also not been quantitatively characterized. In addition, all material characterizations in this work were performed under laboratory-controlled ambient conditions. Limited replicate numbers were available for several performance tests, which may increase uncertainty in some experimental results.

From an application perspective, key parameters relevant to practical toy use, such as surface hardness, flexural properties, and abrasion resistance, remain unexplored, as the current mechanical characterization is restricted to basic material evaluation. In addition, the color stability of dyed samples, including wash fastness and UV aging resistance, has not been evaluated, nor have the antimicrobial properties required for child-contact applications been systematically examined. This is particularly relevant given the biodegradable nature of the materials, as degradation may promote microbial growth under humid conditions—a scenario commonly encountered in children’s play environments. Future work will incorporate antimicrobial testing (e.g., against representative bacterial strains) under relevant conditions to assess the material’s resistance to microbial colonization.

However, this test only captures seed germination and early seedling growth under the tested condition and cannot reflect chronic impacts on soil biota such as earthworms and springtails, or potential shifts in the composition and function of soil microbial communities. Furthermore, leachable substances released during sample incubation may induce sublethal or cumulative toxic effects undetectable in this short-term, unreplicated test. No claims about non-toxicity or environmental safety can be drawn from this exploratory assay.

In addition, quantitative colorimetric characterization of both undyed and dyed samples and toy-relevant toxicological assessments—including migration of regulated elements and compounds under EN 71−3/ASTM F963 protocols [87,88], cytotoxicity, and skin sensitization—have not yet been performed and will be necessary before any claim regarding child-contact use can be made.

The above performance gaps do not indicate material inadequacy but instead reflect the preliminary nature of this material-oriented exploration. Comprehensive validation of both microscopic formation mechanisms and application-oriented service performance will be addressed in subsequent studies, in which physical toy prototypes will be fabricated and systematic testing under simulated practical service conditions will be conducted. Overall, this study provides only preliminary baseline data for conceptual bio-based material exploration, and further systematic optimization and validation are required before practical application.

## 5. Conclusions

This study carried out a preliminary laboratory-scale fabrication and basic performance characterization of two bio-based composite films: corn-starch-based and orange-peel-gelatin-based composites, for exploratory conceptual design of an eco-friendly toy.

Both composite systems could be colored with natural plant pigments to obtain various hues, indicating the feasibility of plant-based dyeing for these biocomposite systems under the tested experimental conditions. Tensile tests showed distinct mechanical behaviors between the two material systems, and their mechanical properties could be adjusted by varying the contents of starch, gelatin, plasticizer, and orange-peel filler. Soil burial testing revealed different mass change profiles for starch and gelatin composites over the test period. Seed germination assays under the tested conditions did not show obvious acute inhibitory effects, but these observations cannot confirm the absence of phytotoxicity.

Based on their respective tensile characteristics, conceptual toy design schemes were tentatively proposed for the two materials. It must be emphasized that the toy-related designs are only theoretical conceptual explorations, without verification of mechanical safety, migration performance, cytotoxicity, and toy-specific safety indicators. Thus, the materials cannot be confirmed to be applicable for formal children’s toy products. Overall, the present work provides baseline experimental data for further development of starch- and gelatin-based biocomposites. All findings reported herein are preliminary. Future studies will focus on quantitative performance testing, safety validation, and interfacial and mechanical analysis to further improve and complement the current understanding of such bio-based composites.

## Figures and Tables

**Table 1:** Type 5A specimen dimensions according to GB/T 1040.2-2022.

Sample Type		5A
l3	Total Length	≥75
b2	End Width	12.5 ± 1
l1	Length of Narrow Parallel Part	25 ± 1
b1	Width of Narrow Part	4 ± 0.1
r1	Small Radius	8 ± 0.5
r2	Large Radius	12.5 ± 1
L	Initial Distance Between Fixtures	50 ± 2
L0	Gauge Length	20 ± 0.5
h	Thickness	2 ± 0.2

Note(s): All dimensions are in millimeters.

## Data Availability

The data supporting the findings of this study are openly available in the Figshare repository at https://doi.org/10.6084/m9.figshare.30742487, accessed on 30 September 2026.

## Appendix A

**Table A1:** Formulation parameters for starch-based composites.

	Glycerol (g)	White Vinegar (g)	Water (mL)	Corn Starch (g)
Sample 1	1	4.2	150	10
	5	4.2	150	10
	9	4.2	150	10
Sample 2	4.2	1	150	10
	4.2	5	150	10
	4.2	9	150	10
Sample 3	4.2	4.2	150	3
	4.2	4.2	150	10
	4.2	4.2	150	7

**Table A2:** Formulation parameters for orange-peel-gelatin-based composites.

	Glycerol (g)	Gelatin (g)	Water (mL)	Orange Peel Powder (g)
Sample 1	6	12	200	8
	12	12	200	8
	18	12	200	8
Sample 2	12	6	200	8
	12	12	200	8
	12	18	200	8
Sample 3	12	12	200	4
	12	12	200	9
	12	12	200	14
